# Comparative efficacy and safety of JAK inhibitors in the treatment of moderate-to-severe alopecia areata: a systematic review and network meta-analysis

**DOI:** 10.3389/fphar.2024.1372810

**Published:** 2024-04-10

**Authors:** Ting Yan, Ting Wang, Mei Tang, Nan Liu

**Affiliations:** ^1^ Department of Pharmacy, Sichuan Provincial People’s Hospital, School of Medicine, University of Electronic Science and Technology of China, Chengdu, Sichuan, China; ^2^ Personalized Drug Therapy Key Laboratory of Sichuan Province, University of Electronic Science and Technology of China, Chengdu, Sichuan, China; ^3^ Departments of Nuclear Medicine, Sichuan Provincial People’s Hospital, School of Medicine, University of Electronic Science and Technology of China, Chengdu, Sichuan, China

**Keywords:** alopecia areata, JAK inhibitors, network meta-analysis, rct, efficacy, safety

## Abstract

We performed a Bayesian network meta-analysis to indirectly compare the relative efficacy and safety of the latest JAK inhibitors for moderate-to-severe alopecia areata (AA). 13 trials totaling 3,613 patients were included. Two low-dose groups of oral formulations (ritlecitinib 10mg and ivarmacitinib 2mg) and two topical formulations (delgocitinib ointment and ruxolitinib cream) appeared to be relatively ineffective against moderate-to-severe AA. Ranking analysis suggested that brepocitinib 30mg has the best relative effect in reducing the SALT score (sucra = 0.9831), and demonstrated comparable efficacy to deuruxolitinib 12mg (sucra = 0.9245), followed by deuruxolitinib 8mg (sucra = 0.7736). Regarding the SALT_50_ response, brepocitinib 30mg ranked highest (sucra = 0.9567), followed by ritlecitinib 50mg (sucra = 0.8689) and deuruxolitinib 12mg (sucra = 0.7690). For achieving the SALT_75_ response, deuruxolitinib 12mg had the highest probability (sucra = 0.9761), followed by deuruxolitinib 8mg (sucra = 0.8678) and brepocitinib 30mg (sucra = 0.8448). Deuruxolitinib 12mg might be the most effective therapy for patients with severe AA (sucra = 0.9395), followed by ritlecitinib 50mg (sucra = 0.8753) and deuruxolitinib 8mg (sucra = 0.8070). Deuruxolitinib 12mg/8mg demonstrated notable efficacy for moderate-to-severe AA, and is expected to be a new treatment option for AA. It was worth noting that deuruxolitinib exhibit a greater likelihood of causing adverse events in comparison to other JAK inhibitors. Ritlecitinib 50mg seemed to exhibit fewer adverse effects in the high-dose groups of oral JAK inhibitors and might be an optimal choice to balance safety and efficacy. The majority of JAK inhibitors exhibited acceptable short-term safety profiles. To enhance the applicability and accuracy of our research, further head-to-head trials with longer follow-up periods are needed.

**Systematic Review Registration:** identifier [CRD42022368012].

## 1 Introduction

Alopecia areata (AA) is a chronic, immune-mediated disease that leads to partial or complete nonscarring hair loss. It can affect individuals of all ages, both females and males, without significant ethnic differences (Gilhar et al., 2012; Strazzulla et al., 2018). The symptoms of AA may spontaneously resolve over time, but it can unpredictably recur after remission and even progress to alopecia totalis (AT), which involves total hair loss of the scalp, or alopecia universalis (AU), which involves hair loss of the entire body (Strazzulla et al., 2018; Simakou et al., 2019).

While AA is typically not life-threatening, the frequent relapses and chronic nature of the condition can contribute to the development of psychological disorders, including depression, anxiety, and suicidal thoughts (Mulinari-Brenner, 2018; Toussi et al., 2021). In addition, traditional treatments for alopecia areata (AA), such as topical or systemic corticosteroids, minoxidil, methotrexate, and azathioprine, exhibit limited efficacy, particularly in moderate to severe cases. These treatments are also associated with a high recurrence rate and an increased incidence of adverse events (Zhou et al., 2021). With further research on the pathogenesis of AA, a variety of promising drugs emerged, offering a new prospect for the treatment of AA. Among these, Janus kinase (JAK) inhibitors are considered to be a better treatment option (Ocampo-Garza et al., 2019; Zhou et al., 2021).

The Janus kinase‒signal transducer and activator of transcription (JAK-STAT) pathway mediates signaling downstream of type I and type II cytokines, involving four JAK kinases: JAK1, JAK2, JAK3, and tyrosine kinase 2 (Tyk2). Dysregulated JAK activity is strongly associated with immune dysregulation of many inflammatory dermatoses, including alopecia areata, atopic dermatitis, vitiligo, psoriasis and others (Damsky and King, 2017; Welsch et al., 2017). Substantial evidence demonstrates that the relevant pathogenesis of AA is thought to be related to the collapse of immune privilege in hair follicles. This immune imbalance is mediated by specific cytokines present around the hair follicles, with IFN-γ and CD8+NKG2D+T cells identified as key factors. Activated CD8+NKG2D+T cells produce IFN-γ via JAK pathways, which leads to exposure of autoantigens and facilitates the autoimmune attack on hair follicles. Furthermore, IFN-γ promotes IL-15 production in hair follicles via JAK1 and JAK2, and IL-15, in turn, stimulates the production of more IFN-γ through JAK1/3 signaling, thereby amplifying the inflammatory response around hair follicles (Zhou et al., 2021; Lensing and Jabbari, 2022).

JAK inhibitors, through their targeting of the JAK-STAT signaling pathways, possess the capability to disrupt the production of inflammation-associated cytokines and exert therapeutic effects in the treatment of AA. On 13 June 2022, the FDA approved baricitinib as the first systemic treatment for severe AA (FDA, 2022). Subsequently, on 23 June 2023, ritlecitinib also received FDA approval for the treatment of severe AA in both adolescents and adults (FDA, 2023). Recent advancements in JAK inhibitors, such as brepocitinib, deuruxolitinib, ivarmacitinib, and ATI-501, have demonstrated the potential for substantial enhancement in clinical outcomes among patients with alopecia areata in both phase 2 and 3 trials. However, due to a lack of head-to-head trials and a dearth of network meta-analyses comparing these interventions, the relative efficacy and safety of these JAK inhibitors remain uncertain. Therefore, this network meta-analysis was conducted to assess the efficacy and safety of current and novel JAK inhibitors for moderate-to-severe alopecia areata, based on the existing randomized controlled trials (RCTs). The aim was to provide clinical recommendations for the treatment of AA.

## 2 Methods

Our study protocol was registered in the International Prospective Register of Systematic Reviews (CRD42022368012). The network meta-analysis was performed in accordance with the Preferred Reporting Items for Systematic Review and Meta-Analysis guidelines for Network Meta-Analysis (PRISMA-NMA).

### 2.1 Information sources and search strategy

We conducted searches in the following databases for relevant English language literature: PubMed (MEDLINE), Embase, the Cochrane Central Register of Controlled Trial (CENTRAL) and Web of Science. The retrieval date ranged from the establishment of the database to 5 March 2024. To ensure maximum sensitivity of the search strategy, we combined search terms such as “alopecia areata,” “JAK inhibitor,” and the names of specific drugs, including both thesaurus terms (MeSH and Emtree terms) and international nonproprietary names. For more detailed information on the search strategies, please refer to Supplementary Table S1.

### 2.2 Outcomes of interest

The Severity of Alopecia Tool (SALT) is a validated tool utilized to assess the clinical severity of alopecia, with scores ranging from 0 (indicating no scalp hair loss) to 100 (representing complete scalp hair loss). Pre- and post-intervention SALT scores were compared to evaluate the effectiveness of the intervention (Olsen et al., 2004). Since the efficacy parameters evaluated in these studies showed inconsistency, the main treatment outcomes assessed in our network meta-analysis included 1): change or percentage change in SALT score from baseline to the end of treatment 2), SALT_50_, which represents the number of patients achieving a 50% improvement in SALT score 3), SALT_75_, indicating the number of patients achieving a 75% improvement in SALT score, and 4) the number of patients who achieved absolute SALT scores of ≤20. The primary safety outcome measured was the incidence rate of adverse events (AE).

### 2.3 Eligibility criteria and selection process

Eligible studies for the systematic review and meta-analysis are required to meet the following inclusion criteria 1): being limited to randomized clinical trials involving patients with moderate-to-severe AA 2), including intervention treatments with any JAK inhibitors compared to placebo or other JAK inhibitors, and 3) reporting at least one relevant efficacy outcome (as mentioned in the previous section). Two researchers (MT and TW) independently screened articles based on their titles, abstracts, and full texts, following the predetermined eligibility criteria. Disagreements were resolved through discussion and consensus with a third researcher (TY).

### 2.4 Data extraction and statistical analysis

For all eligible trials, two researchers (MT and TW) independently extracted the following data: first author, publication years, study design, patient characteristics (sample size, mean age, gender, baseline SALT), interventions, study duration, and reported outcomes. Any disagreements were resolved by another researcher (TY or NL).

We performed a Bayesian multi-treatment comparison using the gemtc package in R software (version 4.2.2). Gibbs sampling, implemented through Just Another Gibbs Sampler (JAGS), was utilized. Model parameters were estimated using a Markov Chain Monte Carlo (MCMC) method. The convergence of the chains was assessed by checking the Gelman-Rubin statistic. Rankings probabilities for the different treatments were determined based on the surface area under the cumulative ranking curve (SUCRA). For each outcome, an intervention with a SUCRA value closer to 100 represents better performance. The treatment effect on clinical outcomes will be represented by odds ratio (OR) [for dichotomous outcomes] and mean differences (MD) [for continuous outcomes] with 95% credible intervals (CrI). For efficacy, an MD less than 0 or an OR greater than 1.0 indicates a favorable outcome for the intervention. Regarding safety outcomes, an OR greater than 1.0 favors the comparator. The selection of the fixed-effects model was based on DIC criteria. The network graph and funnel plot were generated using Stata SE 16.

### 2.5 Assessment of risk of bias

Two researchers (TW, MT) independently assessed the risk of bias for each study using the Cochrane Collaboration’s Risk of Bias tool in Review Manager (RevMan) version5.4. Any discrepancies were resolved through consensus with another researcher (NL or TY). The included RCTs were evaluated based on the following domains: random sequence generation, allocation concealment, blinding of participants and personnel, blinding of outcome assessments, incomplete outcome data, selective reporting, and other source biases.

## 3 Results

### 3.1 Characteristics of included studies

**FIGURE 1:**
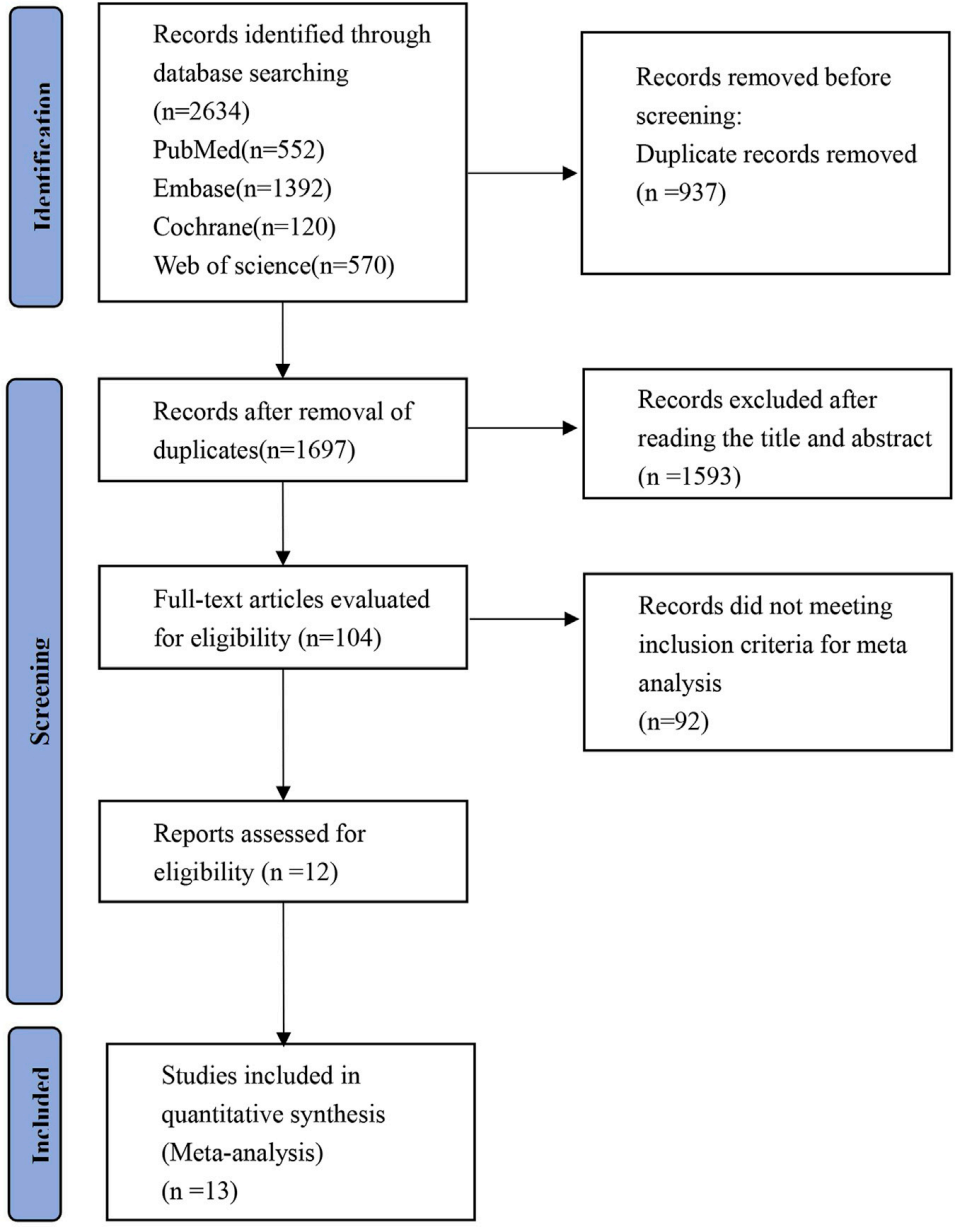
PRISMA flow diagram of the study process. Preferred Reporting Items for Systematic Review and Meta-analysis (PRISMA).

The PRISMA flowchart for the study search is shown in Figure 1. A total of 2,634 records were identified during the systematic search. Following the removal of duplicates, there were 1,697 publications remaining for further evaluation. Subsequently, 1,593 articles were excluded after reviewing their titles and abstracts. Full-text reviews were performed for the remaining 104 articles, and 12 articles comprising 13 RCTs (*n* = 3,613 subjects) fulfilled the inclusion criteria and were finally included in the NMA (King et al., 2021a; King et al., 2021b; King et al., 2022a; King et al., 2022b; King et al., 2023; Mikhaylov et al., 2023; National Library of Medicine, 2020; National Library of Medicine, 2023; National Library of Medicine, 2024a; National Library of Medicine, 2024b; Olsen et al., 2020; Zhou et al., 2023). One publication includes two studies (King et al., 2022b).

The 13 RCTs included two topical JAK inhibitors (delgocitinib ointment and ruxolitinib cream) and six oral JAK inhibitors (ritlecitinib, brepocitinib, baricitinib, ivarmacitinib, ATI-501 and deuruxolitinib). All of these RCTs were placebo-controlled. Among them, 10 studies had both a placebo and at least two active treatment arms, with 9 studies specifically assessing the effects of different dosages of the same drug (ritlecitinib, baricitinib, ivarmacitinib, ATI-501, and deuruxolitinib) (National Library of Medicine, 2020; King et al., 2021b; King et al., 2022a; King et al., 2022b; King et al., 2023; Zhou et al., 2023; National Library of Medicine, 2024a; National Library of Medicine, 2024b) and one study including two different JAK inhibitors (ritlecitinib 50mg and brepocitinib 30mg) (King et al., 2021a). Among all the included RCTs, the average age ranged from 32.1 to 44.3 years, and the proportion of females ranged from 45.5% to 92.6%. The baseline mean SALT score ranged from 53.7 to 92.9, and the mean duration ranged from 12 to 36 weeks. The characteristics of the 13 included studies are presented in Table 1.

**TABLE 1:** Characteristics of the included studies.

Study	Study design	Type of intervention	Sample size	Baseline SALT	Age	Gender (F/M)	Follow-up (week)	Outcome
Mikhaylov 2022 (Mikhaylov et al., 2023) (NCT02561585)	Phase 2	Delgocitinib ointment, twice daily	20	67.19	36.4	14/6	12	①②③⑥
		Placebo	11	74.35	32.1	5/6		
King1 2021 (King et al., 2021a) (NCT02974868)	Phase 2	Ritlecitinib 50mg, once daily	48	89.4	37	37/11	24	①②③④⑥
		Brepocitinib 30mg, once daily	47	86.4	34	32/15		
		Placebo	47	88.4	38	29/18		
King2 2021 (King et al., 2021b) (NCT03570749)	Phase 2	Baricitinib 2mg, once daily	27	86.1	42.5	23/4	36	②③④⑤⑥
		Baricitinib 4mg, once daily	27	83.4	42.4	25/2		
		Placebo	28	90	40.5	16/12		
King3 2022 (King et al., 2022a) (NCT03137381)	Phase 2	Deuruxolitinib 4 mg, twice daily	30	88.8	35.7	22/8	24	③④⑤⑥
		Deuruxolitinib 8 mg, twice daily	38	89.1	37.3	26/12		
		Deuruxolitinib 12 mg, twice daily	37	87.3	35.8	28/9		
		Placebo	44	86.8	37.8	29/15		
Olsen 2020 (Olsen et al., 2020) (NCT02553330)	Phase 2	Ruxolitinib cream, twice daily	39	59.9	44.3	24/15	24	①②③⑥
		Placebo	39	59	42.4	27/12		
King4 2022 (King et al., 2022b) (NCT03570749)	Phase 3	Baricitinib 2mg, once daily	184	86.8	38	109/75	36	①②③④⑤⑥
		Baricitinib 4mg, once daily	281	85.3	36.3	165/116		
		Placebo	189	84.7	37.4	109/80		
King5 2022 (King et al., 2022b) (NCT03899259)	Phase 3	Baricitinib 2mg, once daily	156	85.6	39	103/53	36	①②③④⑤⑥
		Baricitinib 4mg, once daily	234	84.8	38	144/90		
		Placebo	156	85	37.1	98/58		
NCT04517864 (National Library of Medicine, 2023) Allegro2a	Phase 2a	Ritlecitinib 50mg, once daily	36	59.6	35.1	25/11	36	①⑥
		Placebo	35	53.7	34.2	25/10		
King6 2023 (King et al., 2023) (NCT03732807)	phase 2b/3	Ritlecitinib 50mg, once daily	132	90.3	34.5	81/51	24	①④⑤⑥
		Ritlecitinib 30mg, once daily	130	90.5	33.7	85/45		
		Ritlecitinib 10mg, once daily	63	88.3	34.3	43/20		
		Placebo	131	92.9	34	86/45		
Zhou 2023 (Zhou et al., 2023) (NCT04346316)	Phase 2	Ivarmacitinib 2mg, once daily	23	65.2	36	11/12	24	①②③④⑤⑥
		Ivarmacitinib 4mg, once daily	23	62.1	33.3	14/9		
		Ivarmacitinib 8mg, once daily	24	63.9	37.9	15/9		
		Placebo	24	61.3	34.7	12/12		
NCT03594227 (National Library of Medicine, 2020)	Phase 2	ATI-501 400mg, twice daily	23	78	38.7	17/6	24	①②③
		ATI-501 600mg, twice daily	23	76	40.4	12/11		
		ATI-501 800mg, twice daily	22	81	40.5	13/9		
		Placebo	19	85	41.8	14/5		
NCT04518995 (National Library of Medicine, 2024a) (THRIVE-AA1)	Phase 3	Deuruxolitinib 8 mg, twice daily	351	85.5	38.9	217/134	24	②④⑤⑥
		Deuruxolitinib 12 mg, twice daily	215	85.2	38.2	131/84		
		Placebo	140	88.1	38.7	89/51		
NCT04797650 (National Library of Medicine, 2024b) (THRIVE-AA2)	Phase 3	Deuruxolitinib 8 mg, twice daily	258	88.1	38.4	177/81	24	②④⑤⑥
		Deuruxolitinib 12 mg, twice daily	129	86.7	39.7	84/45		
		Placebo	130	88.9	39.7	88/42		

①The change from baseline in Severity of Alopecia Tool (SALT) score.

②The percentage change from baseline in SALT score.

③Proportion of participants who achieved 50% improvement in the SALT score(SALT_50_).

④Proportion of participants who achieved 75% improvement in the SALT score(SALT_75_).

⑤Proportion of participants who achieved absolute SALT score of ≤20.

⑥Adverse event.

### 3.2 Risk of bias

Supplementary Figures S1, S2 depict the assessments of bias risk using the Cochrane Collaboration’s risk of bias tool. All of the studies demonstrated a low to moderate risk of bias in the six domains assessed according to Cochrane criteria. Regarding sequence generation and allocation concealment, the risk of bias for three articles was graded as ‘unclear’ due to the lack of a clear description regarding the methods employed (King et al., 2021b; King et al., 2022a; Mikhaylov et al., 2023).

We classified the risk as “unclear” for 1 trial in terms of “attrition bias” due to the absence of a description regarding the method used to handle missing data (Mikhaylov et al., 2023).

The research data and protocols of four studies were obtained from clinicaltrials.gov, but have not yet been published in peer-review journals. Therefore, we classified these studies as “unclear” in the “other bias” domain (National Library of Medicine, 2020; National Library of Medicine, 2023; National Library of Medicine, 2024a; National Library of Medicine, 2024b).

### 3.3 The change and percentage change from baseline in SALT score

**FIGURE 2:**
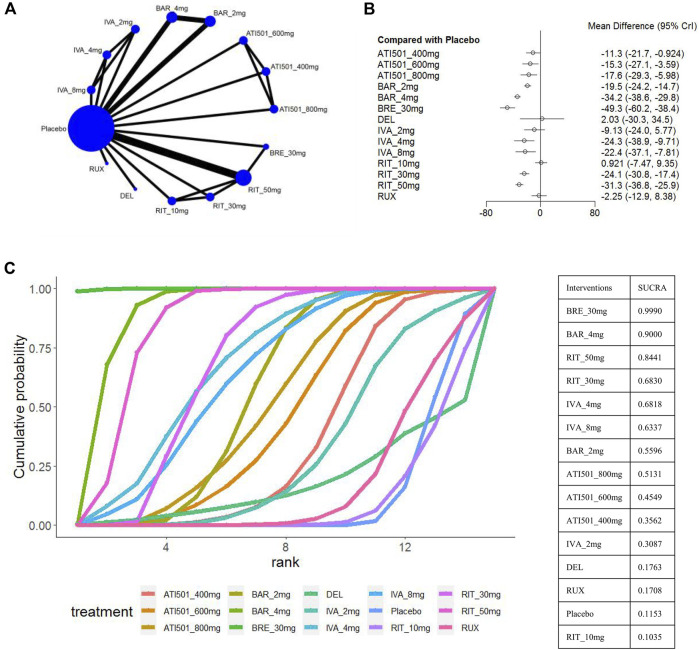
Summary of change in SALT score from baseline to the end of treatment. **(A)** Network diagrams of comparisons, the width of the lines proportional to the number of studies evaluating each direct comparison, and the size of nodes is proportional to the number of participants. **(B)** The forest plot for change in SALT score compared with placebo. Effect sizes are presented as mean differences (MDs) with 95% credible interval (CrI). **(C)** SUCRA-based ranking probabilities graph of each treatments. BRE_30mg: brepocitinib 30mg; BAR_4mg: baricitinib 4mg; BAR_2mg: baricitinib 2mg; RIT_50mg: ritlecitinib 50mg; RIT_30mg: ritlecitinib 30mg; RIT_10mg: ritlecitinib 10mg; IVA_8mg: ivarmacitinib 8mg; IVA_4mg: ivarmacitinib 4mg; IVA_2mg: ivarmacitinib 2mg; RUX: ruxolitinib cream; DEL, delgocitinib ointment.

The change in SALT score was reported in 9 trials (National Library of Medicine, 2020; Olsen et al., 2020; King et al., 2021a; King et al., 2022b; King et al., 2023; Mikhaylov et al., 2023; National Library of Medicine, 2023; Zhou et al., 2023) with a total of 14 treatments, including brepocitinib 30mg, baricitinib 4mg/2mg, ritlecitinib 50mg/30mg/10mg, ivarmacitinib 8mg/4mg/2mg, ATI501 800mg/600mg/400mg, delgocitinib ointment, and ruxolitinib cream (Figure 2A). Except for the two low-dose groups (ritlecitinib 10mg and ivarmacitinib 2mg), all oral formulations were associated with a reduction in SALT score compared to the placebo. The two topical preparations did not show a statistically significant difference from placebo in reducing SALT score (Figure 2B). Based on the ranking analysis, brepocitinib 30 mg exhibited the greatest in the change of SALT score (sucra = 0.9990). This is followed by baricitinib 4mg (sucra = 0.9000), ritlecitinib 50mg (sucra = 0.8441), ritlecitinib 30mg (sucra = 0.6830), ivarmacitinib 4mg (sucra = 0.6818) and ivarmacitinib 8mg (sucra = 0.6337) (Figure 2C).

**FIGURE 3:**
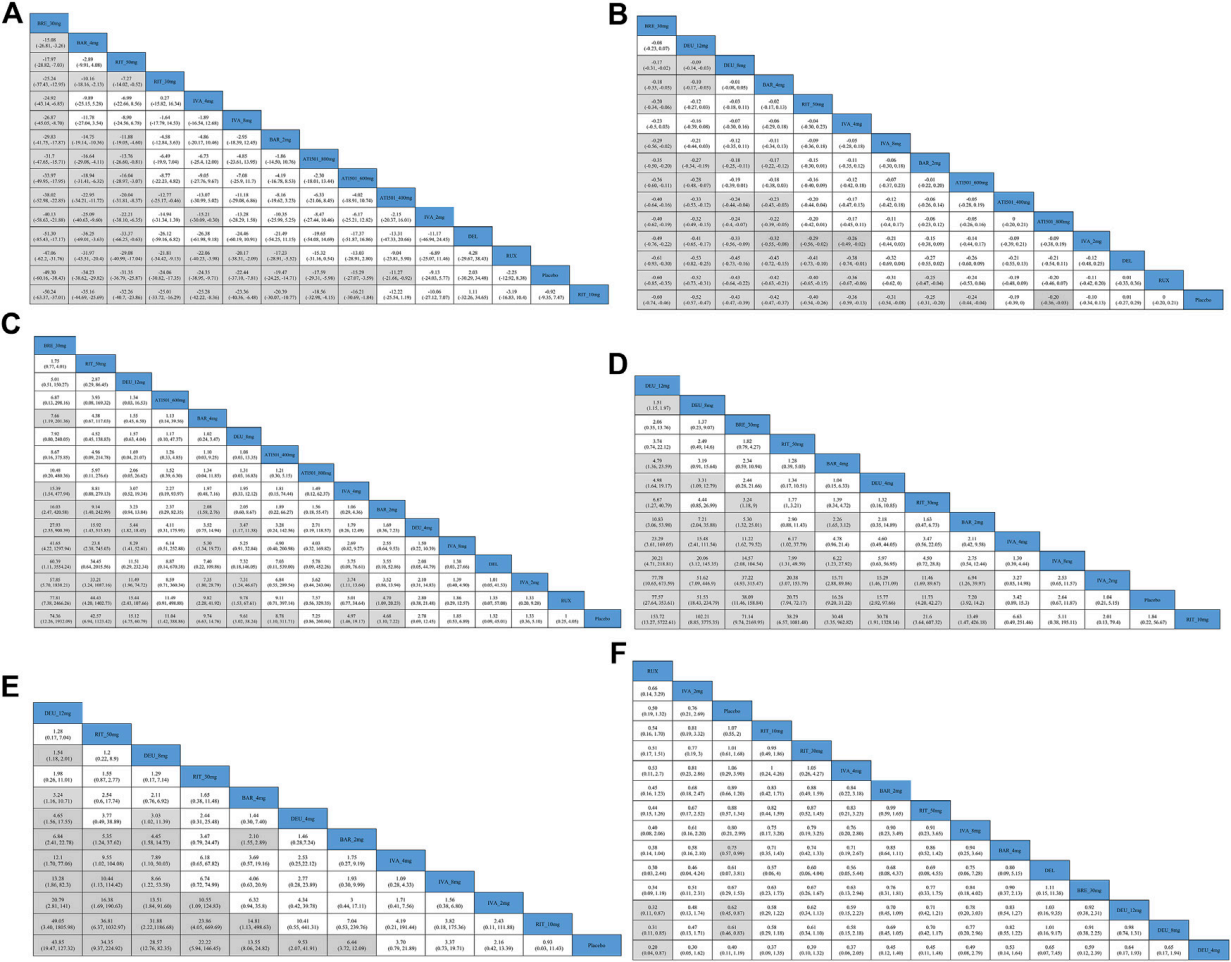
Results of comparisons between each of the interventions for outcome indicators **(A)** the change in SALT score; **(B)** the percentage change in SALT score; **(C)** SALT_50_; **(D)** SALT_75_; **(E)** the percentage of patients who achieve SALT score ≤20; **(F)** AEs (adverse events). BRE_30mg: brepocitinib 30mg; DEU_12mg: deuruxolitinib 12mg; DEU_8mg: deuruxolitinib 8mg; DEU_4mg: deuruxolitinib 4mg; BAR_4mg: baricitinib 4mg; BAR_2mg: baricitinib 2mg; RIT_50mg: ritlecitinib 50mg; RIT_30mg: ritlecitinib 30mg; RIT_10mg: ritlecitinib 10mg; IVA_8mg: ivarmacitinib 8mg; IVA_4mg: ivarmacitinib 4mg; IVA_2mg: ivarmacitinib 2mg; RUX: ruxolitinib cream; DEL, delgocitinib ointment.

The results of the comparisons between each of the drugs are presented in Figure 3A. Brepocitinib 30mg demonstrated superior performance compared to other treatments in terms of change in SALT scores: including baricitinib 4mg (MD −15.08, 95% CI −26.81 to −3.26), ritlecitinib 50mg (MD −17.97, 95% CrI −28.82 to −7.03), ivarmacitinib 4mg (MD −24.92, 95% CrI −43.14 to −6.85); ATI501 800mg(MD −31.70, 95% CrI −47.65 to −15.71), ruxolitinib cream (MD −47.06, 95% CrI −62.20 to −31.76), and delgocitinib ointment (MD −51.30, 95% CrI −85.43 to −17.17). The higher dose groups performed better overall compared to their respective weaker counterparts: baricitinib 4mg versus baricitinib 2mg (MD −14.75, 95% CrI −19.14 to −10.36); ritlecitinib 50mg versus ritlecitinib 30mg (MD −7.27, 95% CrI −14.02 to −0.52). However, no dose-dependent treatment effects were observed in ATI501 or ivarmacitinib. Both baricitinib 4mg and ritlecitinib 50mg demonstrated a similar improvement trend in SALT score change from baseline.

**FIGURE 4:**
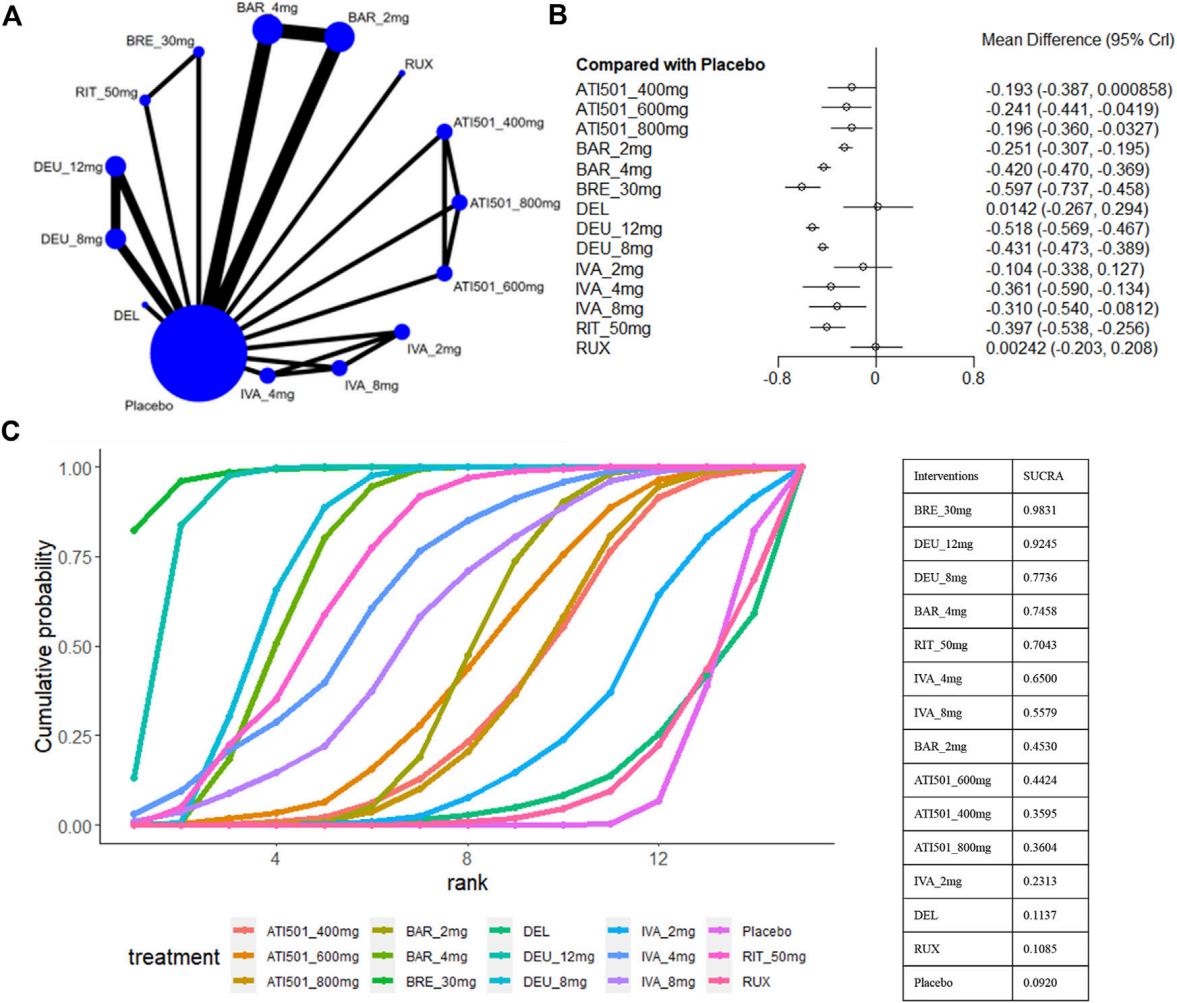
Summary of the percentage change in SALT score from baseline to the end of treatment. **(A)** Network diagrams of comparisons, the width of the lines proportional to the number of studies evaluating each direct comparison, and the size of nodes is proportional to the number of participants. **(B)** The forest plot for the percentage change in SALT score compared with placebo. Effect sizes are presented as mean differences (MDs) with 95% credible interval (CrI). **(C)** SUCRA-based ranking probabilities graph of each treatments. BRE_30mg: brepocitinib 30mg; DEU_12mg: deuruxolitinib 12mg; DEU_8mg: deuruxolitinib 8mg; BAR_4mg: baricitinib 4mg; BAR_2mg: baricitinib 2mg; RIT_50mg: ritlecitinib 50mg; IVA_8mg: ivarmacitinib 8mg; IVA_4mg:ivarmacitinib 4mg; IVA_2mg: ivarmacitinib 2mg; RUX: ruxolitinib cream; DEL: delgocitinib ointment.

The percentage change in SALT score was reported in 10 trials (National Library of Medicine, 2020; Olsen et al., 2020; King et al., 2021a; King et al., 2021b; King et al., 2022b; Mikhaylov et al., 2023; Zhou et al., 2023; National Library of Medicine, 2024a; National Library of Medicine, 2024b), involving brepocitinib 30mg, ritlecitinib 50mg, ivarmacitinib 8mg/4mg/2mg, deuruxolitinib 12mg/8mg, baricitinib 4mg/2mg, ATI501 800mg/600mg/400mg, delgocitinib ointment and ruxolitinib cream (Figure 4A). All interventions, except for ivarmacitinib 2mg, ATI501 400mg, and the two topical formulations, were superior to placebo (Figure 4B). In terms of the relative change in SALT score (Figure 4C), brepocitinib 30mg maintained its top ranking (sucra = 0.9831), followed by deuruxolitinib 12mg (sucra = 0.9245), deuruxolitinib 8mg (sucra = 0.7736), baricitinib 4mg (sucra = 0.7458), ritlecitinib 50mg (sucra = 0.7043), and ivarmacitinib 4mg (sucra = 0.6500). The ranking of ATI501 800mg/600mg/400 mg was relatively lower, which aligns with the ranking of the change in SALT score. In the pairwise comparisons depicted in Figure 3B, brepocitinib 30 mg appeared to be comparable to deuruxolitinib 12mg in terms of the relative change in SALT score and both interventions were superior to baricitinib 4mg: brepocitinib 30mg versus baricitinib 4mg (MD −0.18, 95% CrI −0.33 to −0.03); deuruxolitinib 12mg versus baricitinib 4mg (MD −0.10, 95% CrI −0.17 to −0.03). Deuruxolitinib 8mg demonstrated comparable efficacy to ritlecitinib 50mg and baricitinib 4mg. Furthermore, a dose-dependent superiority was evident between deuruxolitinib 12mg and 8mg: deuruxolitinib 12mg versus deuruxolitinib 8mg (MD −0.09, 95% CrI −0.14 to −0.03).

### 3.4 Proportion of participants who achieved SALT_50_

**FIGURE 5:**
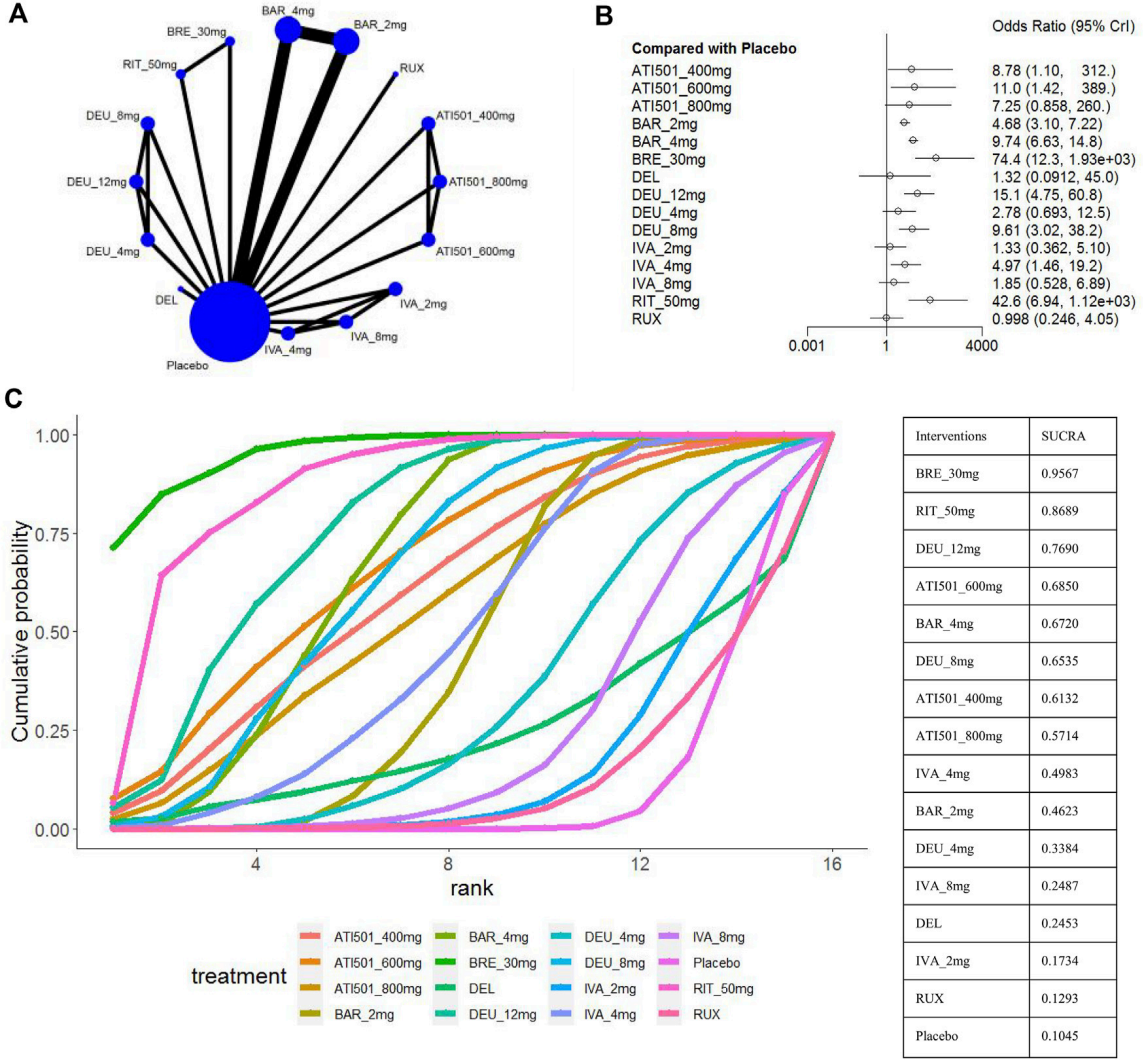
Summary of percentage of patients who achieved 50% improvement in the SALT score. **(A)** Network diagrams of comparisons, the width of the lines proportional to the number of studies evaluating each direct comparison, and the size of nodes is proportional to the number of participants. **(B)** The forest plot for SALT_50_ compared with placebo. Effect sizes are presented as Odds Ratio (OR) with 95% credible interval (CrI). **(C)** SUCRA-based ranking probabilities graph of each treatment. BRE_30mg: brepocitinib 30mg; DEU_12mg: deuruxolitinib 12mg; DEU_8mg:deuruxolitinib 8mg; DEU_4mg:deuruxolitinib 4mg; BAR_4mg: baricitinib 4mg; BAR_2mg: baricitinib 2mg; RIT_50mg: ritlecitinib 50mg; IVA_8mg: ivarmacitinib 8mg; IVA_4mg: ivarmacitinib 4mg; IVA_2mg: ivarmacitinib 2mg; RUX: ruxolitinib cream; DEL, delgocitinib ointment.

Four studies were excluded from the estimation as they did not report the SALT_50_ outcome (King et al., 2023; National Library of Medicine, 2023; National Library of Medicine, 2024a; National Library of Medicine, 2024b) (Figure 5A). Nine interventions were found to be more effective than placebo in terms of reaching SALT_50_. Among the included interventions groups, deuruxolitinib 4mg, ATI501 800mg, ivarmacitinib 2mg/8mg and two topical JAK inhibitors (delgocitinib ointment and ruxolitinib cream) did not show significantly superior efficacy compared to placebo in terms of reaching SALT_50_ (Figure 5B). Ranking analysis indicated that patients receiving brepocitinib 30mg had the highest likelihood of achieving SALT_50_ (sucra = 0.9567), followed by ritlecitinib 50mg (sucra = 0.8689), deuruxolitinib 12mg (sucra = 0.7690), ATI501 600mg (sucra = 0.6850), baricitinib 4mg (sucra = 0.6720), and deuruxolitinib 8mg (sucra = 0.6535) (Figure 5C). No significant difference was observed among the top three ranked interventions (brepocitinib 30mg, ritlecitinib 50mg and deuruxolitinib 12mg). Baricitinib 4mg still achieved a higher SALT_50_ response rate compared to baricitinib 2mg (OR 2.08, 95% CrI 1.58–2.76) (Figure 3C).

### 3.5 Proportion of participants who achieved SALT_75_

**FIGURE 6:**
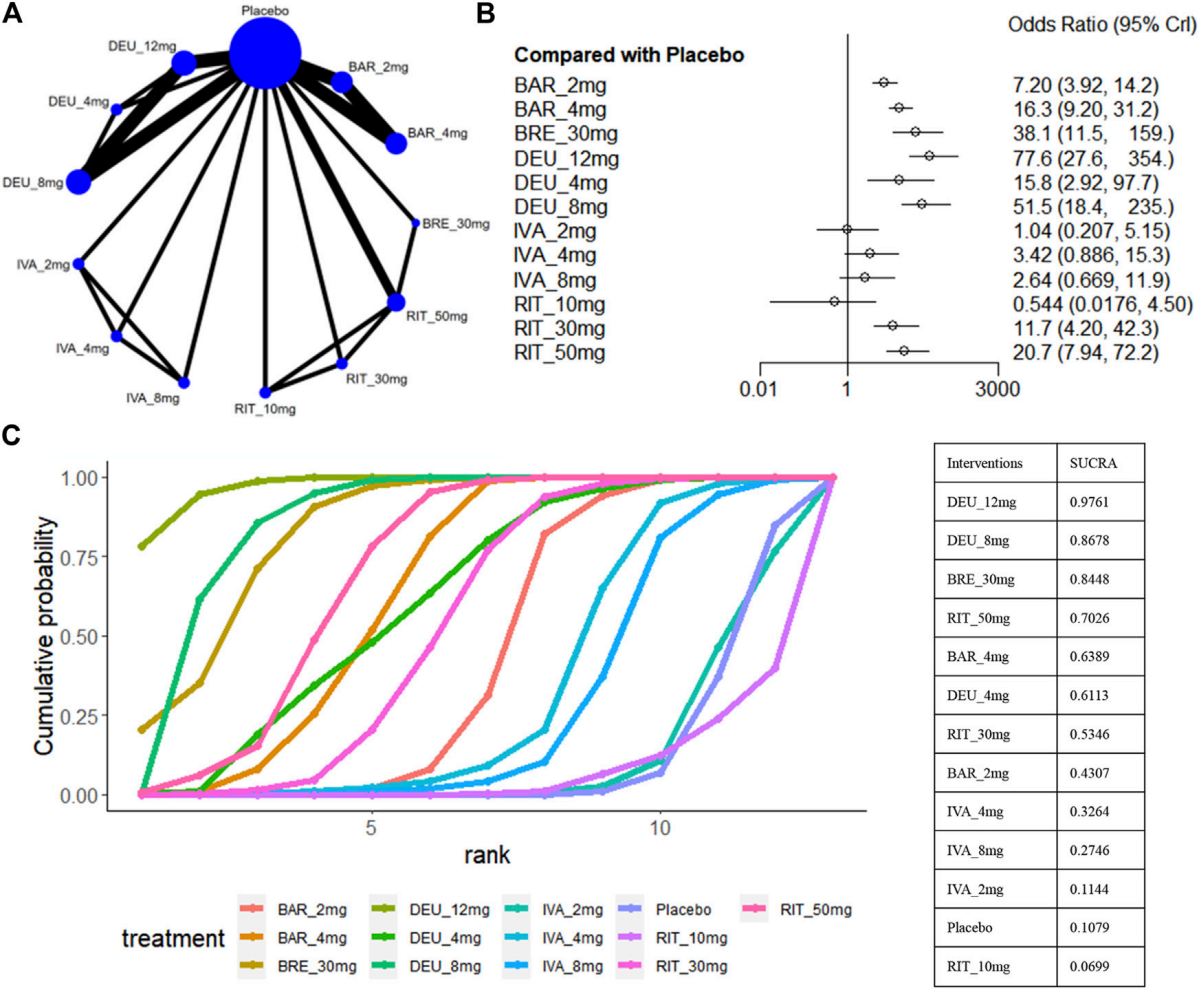
Summary of percentage of patients who achieved 75% improvement in the SALT score. **(A)** Network diagrams of comparisons. The width of the lines proportional to the number of studies evaluating each direct comparison, and the size of nodes is proportional to the number of participants. **(B)** The forest plot for SALT_75_ compared with placebo. Effect sizes are presented as Odds Ratio (OR) with 95% credible interval (CrI). **(C)** SUCRA-based ranking probabilities graph of each treatment. BRE_30mg: brepocitinib 30mg; DEU_12mg: deuruxolitinib 12mg; DEU_8mg: deuruxolitinib 8mg; DEU_4mg: deuruxolitinib 4mg; BAR_4mg: baricitinib 4mg; BAR_2mg: baricitinib 2mg; RIT_50mg: ritlecitinib 50mg; RIT_30mg: ritlecitinib 30mg; RIT_10mg: ritlecitinib 10mg; IVA_8mg: ivarmacitinib 8mg; IVA_4mg: ivarmacitinib 4mg; IVA_2mg: ivarmacitinib 2mg.

The SALT_75_ outcome was available in nine trials (King et al., 2021a; King et al., 2021b; King et al., 2022a; King et al., 2022b; King et al., 2023; Zhou et al., 2023; National Library of Medicine, 2024a; National Library of Medicine, 2024b), involving brepocitinib 30mg, deuruxolitinib 12mg/8mg/4mg, ritlecitinib 50mg/30mg/10mg, baricitinib 4mg/2mg and ivarmacitinib 8mg/4mg/2mg (Figure 6A). When compared with placebo, ritlecitinib 10mg and ivarmacitinib 8mg/4mg/2mg did not reach statistical significance in achieving SALT_75_ (Figure 6B). Cumulative ranking probabilities indicate that deuruxolitinib 12mg appears to be the most efficacious drug in this parameter (sucra = 0.9761), followed by deuruxolitinib 8mg (sucra = 0.8678), brepocitinib 30mg (sucra = 0.8448), ritlecitinib 50mg (sucra = 0.7026), and baricitinib 4mg (sucra = 0.6389) (Figure 6C). Deuruxolitinib 12mg showed superiority over deuruxolitinib 8mg (OR 1.51, 95% CrI 1.15–1.97) and baricitinib 4mg (OR 4.79, 95% CrI 1.36–23.59) in achieving SALT_75_, however, there was no significant difference between deuruxolitinib 12mg, brepocitinib 30mg and ritlecitinib 50mg in this measured parameter. The three dosage groups of ivarmacitinib exhibited lower efficacy compared to other JAK inhibitor groups in achieving SALT_75_ (Figure 3D).

### 3.6 Proportion of participants who achieved absolute SALT scores of ≤20

**FIGURE 7:**
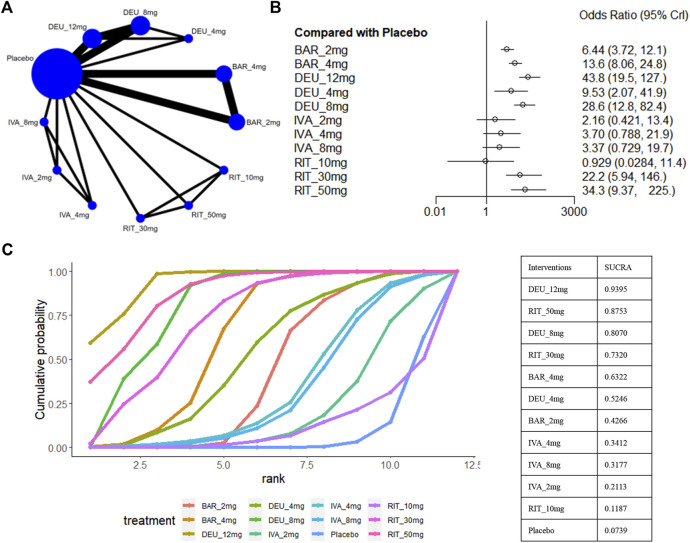
Summary of the percentage of patients who achieved SALT score ≤20. **(A)** Network diagrams of comparisons. The width of the lines proportional to the number of studies evaluating each direct comparison, and the size of nodes is proportional to the number of participants. **(B)** The forest plot for percentage of patients Who Achieve SALT score ≤20 compared with placebo. Effect sizes are presented as Odds Ratio (OR) with 95% credible interval (CrI). **(C)** SUCRA-based ranking probabilities graph of each treatment. DEU_12mg: deuruxolitinib 12mg; DEU_8mg: deuruxolitinib 8mg; DEU_4mg: deuruxolitinib 4mg; BAR_4mg: baricitinib 4mg; BAR_2mg: baricitinib 2mg; RIT_50mg: ritlecitinib 50mg; RIT_30mg: ritlecitinib 30mg; RIT_10mg: ritlecitinib 10mg; IVA_8mg: ivarmacitinib 8mg; IVA_4mg: ivarmacitinib 4mg; IVA_2mg: ivarmacitinib 2mg.

A SALT score of 20 or lower has been identified as a significant treatment outcome for patients with severe AA (severe AA defined as a SALT score of ≥50) (Wyrwich et al., 2020). Eight studies reported these efficacy parameters in a population with severe AA (King et al., 2021b; King et al., 2022a; King et al., 2022b; King et al., 2023; National Library of Medicine, 2024a; National Library of Medicine, 2024b; Zhou et al., 2023), involving ritlecitinib 50mg/30mg/10mg, ivarmacitinib 8mg/4mg/2mg, deuruxolitinib 12mg/8mg/4mg, and baricitinib 4mg/2mg (Figure 7A). For this outcome, we found that ivarmacitinib 8mg/4mg/2mg and ritlecitinib 10mg were relatively ineffective against severe AA (Figure 7B). The NMA revealed that deuruxolitinib 12mg (sucra = 0.9395), ritlecitinib 50mg (sucra = 0.8753), deuruxolitinib 8mg (sucra = 0.8070), ritlecitinib 30mg (sucra = 0.7320) were higher ranked in the treatment of severe AA (Figure 7C).

Based on the comparisons between the drugs, deuruxolitinib 12mg appeared comparable to ritlecitinib 50mg in terms of the proportion of patients achieving a SALT score≤20. Furthermore, deuruxolitinib 12mg demonstrated superior effectiveness compared to deuruxolitinib 8mg (OR 1.54, 95% CrI 1.18–2.01) and baricitinib 4mg (OR 3.24, 95% CrI 1.16–10.71) (Figure 3E).

### 3.7 The proportions of participants with adverse events

**FIGURE 8:**
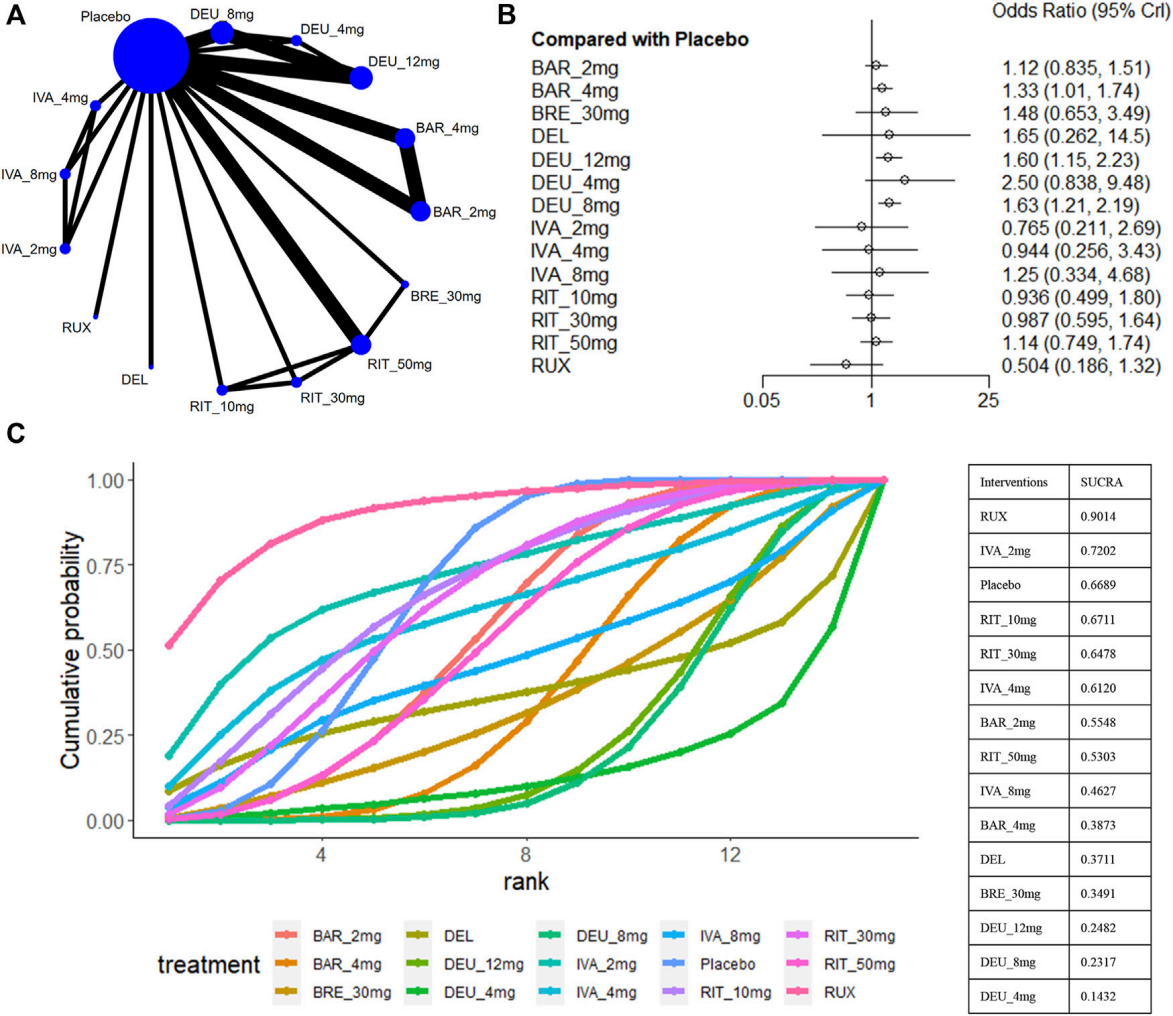
Summary of adverse events. **(A)** Network diagrams of comparisons, the width of the lines proportional to the number of studies evaluating each direct comparison, and the size of nodes is proportional to the number of participants. **(B)** The forest plot for AE compared with placebo. Effect sizes are presented as Odds Ratio (OR) with 95% credible interval (CrI). **(C)** SUCRA-based ranking probabilities graph of each treatments. BRE_30mg: brepocitinib 30mg; DEU_12mg:deuruxolitinib 12mg; DEU_8mg:deuruxolitinib 8mg; DEU_4mg:deuruxolitinib 4mg; BAR_4mg: baricitinib 4mg; BAR_2mg: baricitinib 2mg; RIT_50mg:ritlecitinib 50mg; RIT_30mg:ritlecitinib 30mg; RIT_10mg: ritlecitinib 10mg; IVA_8mg:ivarmacitinib 8mg; IVA_4mg:ivarmacitinib 4mg; IVA_2mg:ivarmacitinib 2mg; RUX:ruxolitinib cream; DEL: delgocitinib ointment.

For safety outcomes, a total of 12 studies reported the data of AE (King et al., 2021a; King et al., 2021b; King et al., 2022a; King et al., 2022b; King et al., 2023; Mikhaylov et al., 2023; National Library of Medicine, 2023; National Library of Medicine, 2024a; National Library of Medicine, 2024b; Olsen et al., 2020; Zhou et al., 2023) (Figure 8A). A comparative analysis of the safety profile for ATI-501 was not possible due to the absence of relevant data. Baricitinib 4mg (OR 1.33, 95% CrI 1.01–1.74), deuruxolitinib 8mg (OR 1.63, 95% CrI 1.21–2.19) and deuruxolitinib 12mg (OR 1.60, 95% CrI 1.15–2.23) were linked to a higher incidence of adverse events (AEs) compared to the placebo, whereas none of the other interventions showed an increased risk of AEs (Figure 8B).

Ranking analysis suggests that ruxolitinib cream was well-tolerated and associated with a minimal risk of side effects (sucra = 0.9014), while deuruxolitinib 4mg/8mg/12mg ranked last (Figure 8C). In general, lower-dose groups achieved higher safety rankings compared to their respective higher-dose groups, except for deuruxolitinib. Ritlecitinib 50mg was ranked higher than the other high-dose intervention groups, but no notable differences in the risk of AEs were observed among these JAK inhibitors (Figure 3F).

In topical formulations, the prevalent adverse effects were local reactions, including folliculitis, conjunctivitis, dry skin, and pruritus. Common adverse effects reported with oral JAK inhibitors primarily included acne, upper respiratory tract infections, headache, urinary tract infection, elevated creatine kinase levels, herpes zoster, and folliculitis. These effects were mostly of mild to moderate severity. We did not summarize specific adverse events since the classification varied across different data sources. Additionally, comparisons of serious adverse events (SAEs) were not feasible due to the limited number of events.

### 3.8 Publication bias assessment

Publication bias was assessed for various intervention combinations in relation to these parameters. Each dot represents the included studies, with different colors indicating different interventions. The comparison-correction funnel chart suggests the presence of potential publication bias. Supplementary Figure S3 provides detailed results of the publication bias assessments.

## 4 Discussions

In a number of recent clinical trials, JAK inhibitors have shown promising results in the treatment of patients with AA. We collected and analyzed the available data to determine the relative efficacy and safety of various JAK inhibitors used in the treatment of moderate-to-severe AA. Our meta-analysis is based on 13 RCTs, including two topical JAK inhibitors and six oral JAK inhibitors with varying dosages.

The oral JAK inhibitors investigated in our study (brepocitinib, ritlecitinib, baricitinib, deuruxolitinib, ivarmacitinib, and ATI501) were proven effective in promoting hair regrowth for moderate-to-severe AA, with the exception of ivarmacitinib 2mg and ritlecitinib 10mg. However, the two topically applied JAK inhibitors (delgocitinib ointment and ruxolitinib cream) seemed to have no effect on patients with moderate-to-severe AA, aligning with findings from previous studies (Phan and Sebaratnam, 2019; Yan et al., 2022; Wei et al., 2023). Local delivery was designed to reduce the potential side effects of systemic administrations. The delgocitinib ointment and ruxolitinib cream have been successfully used for the treatment of atopic dermatitis and vitiligo (Kim et al., 2020; Nakagawa et al., 2020; Rosmarin et al., 2020; Yagi et al., 2021). Unfortunately, these two topical formulations did not demonstrate promising efficacy in RCTs for AA, possibly attributable to inadequate penetration. Enhancing the penetration of the formulation or developing new targeted delivery systems could prove to be an effective approach to unlock the full potential of topical treatment (Bayart et al., 2017; Christmann et al., 2020).

Baricitinib primarily targets the JAK1 and JAK2 subtypes, becoming the first oral JAK inhibitor approved by the FDA for treating severe AA based on BRAVE-AA1 and BRAVE-AA2 results (FDA, 2022). The recommended starting dosage of baricitinib for patients is 2mg orally daily. Our network meta-analysis supports that both baricitinib 4mg and 2mg demonstrate good efficacy in treating moderate-to-severe AA, with baricitinib 4 mg observed to be superior to 2mg in various efficacy indicators. Moreover, in extension trials of the two RCTs, researchers found that increasing the dose to 4mg significantly improved the response rate for patients who were ineffective with 2mg. Therefore, increasing the dose to 4mg becomes an effective clinical strategy for non-responsive patients to the 2mg dose (Ko et al., 2023). However, it is worth noting that the incidence of adverse events tended to increase with the dosing boost.

The increasing comprehension of individual JAK isoforms and their distinct impacts on AA has led to the creation of more JAK-specific compounds. Recent studies indicate that JAK1 and JAK3 play equally crucial roles in the pathogenesis of AA, whereas JAK2 signaling does not. This underscores that selectively targeting JAK1 or JAK3 could enhance the precision of AA therapy and reduce the incidence of hematologic adverse effects associated with JAK2 inhibition, such as anemia, thrombocytopenia, and neutropenia (Dai et al., 2021; Dai et al., 2022; Mattsson et al., 2023).

ATI-501 is a potent, highly selective inhibitor of JAK1 and JAK3 (National Library of Medicine, 2020). The ranking of each dosage group of ATI-501 was comparatively lower in the reduction of SALT, and they did not demonstrate any apparent advantage over other oral JAK inhibitors in treating moderate-to-severe AA. Ivarmacitinib, a highly selective JAK1 inhibitor, is currently undergoing clinical development for autoimmune diseases such as atopic dermatitis and ulcerative colitis (Zhou et al., 2023). The existing data analysis indicates that ivarmacitinib 2mg is not effective for moderate-to-severe AA, possibly due to the failure to achieve therapeutic concentrations. Although ivarmacitinib at doses of 8mg and 4mg showed statistically significant differences compared to the placebo in the reduction of SALT score, neither ivarmacitinib 8mg nor 4mg demonstrated any advantages over the placebo in achieving SALT_75_, and they were also relatively ineffective against severe AA. Moreover, the ranking probability for efficacy among different doses of ATI-501 or ivarmacitinib was not proportional to dose. This lack of dose-dependent response may be attributed to a plateau effect or the small sample size (as only one small-sample study of ivarmacitinib and ATI-501 was included in the analysis). More studies are needed to verify the effectiveness of ivarmacitinib and ATI-501 in the treatment of alopecia areata and to explore the relationship between different doses and therapeutic efficacy to determine the optimal therapeutic dosage.

Based on the ranking of reduction in SALT scores, brepocitinib 30mg emerged as potentially the most effective therapy for moderate-to-severe AA. It also ranks highly in both SALT_50_ and SALT_75_ responses. Brepocitinib is a TYK2/JAK1 inhibitor that has demonstrated therapeutic efficacy in psoriatic arthritis and ulcerative colitis (Fensome et al., 2018; Mease et al., 2023; Sandborn et al., 2023). Existing literature has limited capacity to investigate the efficacy and safety of brepocitinib in managing alopecia areata. Only one published Phase 2 RCT included the use of brepocitinib 30mg and ritlecitinib 50mg in the comparison, potentially affecting the stability and accuracy of the results. Further clinical studies for the treatment of alopecia areata did not proceed with brepocitinib due to the occurrence of two severe cases of rhabdomyolysis events observed during this Phase 2 trial.

Ritlecitinib, a covalent dual kinase inhibitor with high selectivity for JAK3 and the TEC kinase family, has received FDA approval for the treatment of severe alopecia areata in adults and adolescents aged 12 years and older (Xu et al., 2019; FDA, 2023). The tyrosine kinase expressed in hepatocellular carcinoma (TEC) family of protein kinases is a non-receptor protein tyrosine kinase (PTK). They have a close association with the development, differentiation, and function of B and T cells, while also playing a role in regulating the signaling pathways involved in various immune and inflammatory processes (Mano et al., 1996; Shen et al., 2022). Preclinical data have indicated that the inhibition of the cytolytic function of CD8^+^ T cells and NK cells induced by ritlecitinib is primarily driven by the inhibition of TEC kinase rather than JAK3 (Xu et al., 2019). The dual inhibition of JAK3 and TEC kinases can block multiple inflammatory signaling pathways, reduce hair follicle inflammation and sensitivity, and achieve a stronger and broader intervention in the pathogenesis of AA. Our study revealed that both ritlecitinib 50mg and 30mg exhibited favorable efficacy in moderate-to-severe AA, particularly in the severe cases. Additionally, in a subgroup study of NCT02974868, Guttman-Yassky et al. (2022) found that ritlecitinib 50mg showed greater improvement than brepocitinib 30mg at week 24 in the changes in molecular scalp profiles. Since changes in molecular scalp profiles occur earlier than clinical response, they speculated that the clinical responses to ritlecitinib 50mg will exceed those of brepocitinib 30mg at later time points.

Deuteration was evaluated as a strategy to optimize the pharmacokinetics of existing drugs, aiming to improve their pharmacokinetics and toxicity by modifying their metabolism (Chandra Mouli et al., 2023). Deuruxolitinib is a deuterated form of ruxolitinib that selectively inhibits JAK1 and JAK2 (King et al., 2022a). The incorporation of deuterium in ruxolitinib avoids extensive oxidative metabolism around the cyclopentyl ring, providing more durable JAK inhibition (Di Martino et al., 2023). The findings from our network meta-analysis suggested that deuruxolitinib 12mg consistently held high rankings in various outcome measures, and was superior to baricitinib 4 mg in the reduction of SALT score and the SALT_75_ response. Deuruxolitinib 8mg also demonstrated a favorable treatment response in AA. Deuruxolitinib 12mg and 8mg could potentially be the most effective treatment for severe AA, as a higher proportion of patients with severe AA treated with deuruxolitinib 12mg and 8mg achieved a SALT score ≤20. Our network meta-analysis highlighted that deuruxolitinib has shown significant potential to treat moderate-to-severe AA and is expected to be a new treatment option for patients with alopecia areata.

Regarding safety, only the use of baricitinib 4mg, deuruxolitinib 12mg and deuruxolitinib 8 mg exhibited an odds ratio higher than placebo with a statistically significant difference. The ranking probability based on SUCRA indicated that the topical formulation of ruxolitinib showed the fewest adverse effects. The lower dosage group of oral JAK inhibitors demonstrated comparatively fewer adverse effects. However, the adverse reactions of deuruxolitinib appear to have no dose-dependent relationship. In comparison to other JAK inhibitors, the three dose groups of deuruxolitinib seemed to exhibit a higher propensity to generate adverse events. Ritlecitinib 50 mg was ranked highest in these high-dose groups of oral JAK inhibitors, but no statistically significant difference was observed between these high-dose intervention groups. Available data suggested that these JAK inhibitors included were well tolerated with acceptable short-term safety profiles.

Unlike previously published articles (Phan and Sebaratnam, 2019; Yan et al., 2022; Barati Sedeh et al., 2023; Wei et al., 2023), we compared the relative effectiveness and safety of current JAK inhibitors in patients with moderate-to-severe alopecia areata using a bayesian network meta-analysis. Our study was based on higher quality randomized controlled trials and included more new JAK inhibitors for analysis. In the absence of head-to-head trials, we also made a clear ranking of these JAK inhibitors with their different dosages, providing a reference for clinical decision-making and subsequent relevant clinical studies. Compared to a recently published network meta-analysis (Husein-ElAhmed and Husein-ElAhmed, 2024), our inclusion criteria differed. Our study exclusively focused on various JAK inhibitors and was limited to patients with moderate-to-severe alopecia areata. Additionally, some unpublished data were included in our analysis, which helps to reduce publication bias and increase the number of sample, potentially improving the stability and accuracy of the outcomes. This meta-analysis has several limitations. Firstly, our study encompassed oral and topical JAK inhibitors that may increased heterogeneity. Some drugs had a limited number of studies. The small sample size and insufficient reporting data restrict the statistical power of the analysis, which is evident from the relatively wide 95% CI values observed across the analyses. Secondly, despite the absence of global inconsistency based on DIC, we did not conduct an analysis of node inconsistency due to the presence of only a few closed loops comprising a small number of studies within each NMA network. There may still be undetectable inconsistencies in the network, which can potentially impact the accuracy and stability of the results. Additionally, the present analysis is confined to outcomes observed within the 12–36 weeks timeframe. Considering the high likelihood of AA recurrence and the importance of evaluating the long-term safety of treatment, further trials and extended follow-up periods are necessary to thoroughly investigate the efficacy and safety of JAK inhibitors over the long term.

## 5 Conclusion

Our analysis suggests that deuruxolitinib 12mg/8mg, ritlecitinib 50mg/30mg and baricitinib 4mg/2mg have a clear role in patients with moderate-to-severe AA. Integrated across various indicators, deuruxolitinib 12mg/8mg showed superiority over the other JAK inhibitors in the treatment of AA, especially for severe cases. It may providing a new option for clinical treatment. However, it was worth noting that deuruxolitinib seems to have higher adverse effects compared to other highly selective JAK inhibitors. According to the comprehensive analysis of safety and efficacy results, ritlecitinib 50mg might be the better compromise between efficacy and acceptability. High doses have a stronger effect compared to low doses but may also carry a higher risk of adverse reactions. Therapeutic decisions for patients with moderate-to-severe AA are complex and require considerations of treatment efficacy, safety, and the personal condition of the patient. Our study could provide some references for clinical decision-making. As relevant research is still ongoing and certain clinical trial data are not yet accessible, it is crucial to continue gathering new evidence to enhance the applicability and accuracy of our research.

## Data Availability

The datasets presented in this study can be found in online repositories. The names of the repository/repositories and accession number(s) can be found in the article/Supplementary Material.
