# Supplementary material for: Comparative efficacy and safety of JAK inhibitors in the treatment of moderate-to-severe alopecia areata: a systematic review and network meta-analysis
Source: Front Pharmacol. 2024 Apr 10;15:1372810. doi: 10.3389/fphar.2024.1372810 (PMC11039836; doi:10.3389/fphar.2024.1372810)
Supplement: Supplementary file 1 [file DataSheet1.docx]

**Supplementary materials**

**Supplementary Table 1** Search strategies

***PubMed***

#1 "Janus Kinase Inhibitors"[Mesh]

#2 ((((((((((((Janus Kinase Inhibitors) OR (Janus Kinase Inhibitor)) OR (Inhibitors, Janus Kinase)) OR (Kinase Inhibitors, Janus)) OR (JAK Inhibitors)) OR (Inhibitors, JAK)) OR (Inhibitor, Janus Kinase)) OR (Kinase Inhibitor, Janus)) OR (JAK Inhibitor)) OR (Inhibitor, JAK)) OR (JAK1)) OR (JAK2)) OR (JAK3) OR (TYK2)

#3 ((abrocitinib) OR (PF-04965842)) OR (PF04965842)

#4 ((ritlecitinib) OR (PF-06651600)) OR (PF06651600)

#5 ((tofacitinib) OR (CP-690550)) OR (CP690550)

#6 ((baricitinib) OR (LY3009104)) OR (LY-3009104)

#7 ((ruxolitinib) OR (INCB018424)) OR (INCB-018424)

#8 ((upadacitinib) OR (ABT-494)) OR (ABT494)

#9 ((brepocitinib) OR (PF-06700841)) OR (PF06700841)

#10 ((((fedratinib) OR (SAR302503)) OR (SAR-302503)) OR (TG101348)) OR (TG-101348)

#11 ((filgotinib) OR (GLPG-0634)) OR (GLPG0634)

#12 ((pacritinib) OR (SB1518)) OR (SB-1518)

#13 ((delgocitinib) OR (JTE-052)) OR (JTE052)

#14 ((peficitinib) OR (ASP015K)) OR (JNJ-54781532) OR (JNJ54781532)

#15 ((oclacitinib) OR (PF-03394197)) OR (PF03394197)

#16 ((nezulcitinib) OR (TD-0903)) OR (TD0903)

#17 ((deuruxolitinib) OR (CTP-543)) OR (CTP543)

#18 ((((Ivarmacitinib) OR (SHR0302)) OR (ARQ-255) OR (ARQ255) ) OR (ARQ-252)) OR (ARQ252)

#19 jaktinib

#20 ((ATI-501) OR (ATI501)) OR (ATI-502) OR (ATI502)

#21 #1 OR #2 OR #3 OR #4 OR #5 OR #6 OR #7 OR #8 OR #9 OR #10 OR #11 OR #12 OR #13 OR #14 OR #15 OR #16 OR #17 OR #18 OR #19 OR #20

#22 "Alopecia Areata"[Mesh]

#23 (((((((alopecia areata ) OR (alopecia)) OR (areata)) OR (alopecia totalis)) OR (alopecia universalis)) OR (Hair Loss)) OR (Hair Losses)) OR (patchy hair loss) OR (Alopecia Circumscripta)

#24 #22 OR #23

#25 #21 AND #24

***EMBASE***

#1 'janus kinase inhibitor'/exp

#2 'janus kinase inhibitors' OR 'janus kinase inhibitor' OR 'inhibitors, janus kinase' OR 'kinase inhibitors, janus' OR 'jak inhibitors' OR 'inhibitors, jak' OR 'inhibitor, janus kinase' OR 'kinase inhibitor, janus' OR 'jak inhibitor' OR 'inhibitor, jak' OR 'jak1' OR 'jak2' OR 'jak3' OR 'tyk2'

#3 'abrocitinib' OR 'pf-04965842' OR 'pf04965842'

#4 'ritlecitinib' OR 'pf-06651600' OR 'pf06651600'

#5 'tofacitinib' OR 'cp-690550' OR 'cp690550'

#6 'baricitinib' OR 'ly3009104' OR 'ly-3009104'

#7 'ruxolitinib' OR 'incb018424' OR 'incb-018424'

#8 'upadacitinib' OR 'abt-494' OR 'abt494'

#9 'brepocitinib' OR 'pf-06700841' OR 'pf06700841'

#10 'fedratinib' OR 'sar302503' OR 'sar-302503' OR 'tg101348' OR 'tg-101348'

#11 'filgotinib' OR 'glpg-0634' OR 'glpg0634'

#12 'pacritinib' OR 'sb1518' OR 'sb-1518'

#13 'delgocitinib' OR 'jte-052' OR 'jte052'

#14 'peficitinib' OR 'asp015k' OR 'jnj-54781532' OR 'jnj54781532'

#15 'oclacitinib' OR 'pf-03394197' OR 'pf03394197'

#16 'nezulcitinib' OR 'td-0903' OR 'td0903'

#17 'deuruxolitinib' OR 'ctp-543' OR 'ctp543'

#18 'ivarmacitinib' OR 'shr0302' OR 'arq-255' OR 'arq255' OR 'arq-252' OR 'arq252'

#19 'jaktinib'

#20 'ATI-501' OR 'ATI501' OR 'ATI-502' OR 'ATI502'

#21 #1 OR #2 OR #3 OR #4 OR #5 OR #6 OR #7 OR #8 OR #9 OR #10 OR #11 OR #12 OR #13 OR #14 OR #15 OR #16 OR #17 OR #18 OR #19 OR #20

#22 'alopecia areata'/exp

#23 'alopecia areata' OR 'alopecia' OR 'areata' OR 'alopecia totalis' OR 'alopecia universalis' OR 'hair loss' OR 'hair losses' OR 'patchy hair loss' OR 'alopecia circumscripta'

#24 #22 OR #23

#25 #21 AND #24

***Cochrane Library***

#1 MeSH descriptor: [Janus Kinase Inhibitors] explode all trees

#2 (Janus Kinase Inhibitors):ti,ab,kw OR (Janus Kinase Inhibitor):ti,ab,kw OR (Inhibitors, Janus Kinase):ti,ab,kw OR (Kinase Inhibitors, Janus):ti,ab,kw OR (JAK Inhibitors):ti,ab,kw

#3 (Inhibitor, Janus Kinase):ti,ab,kw OR (Kinase Inhibitor, Janus):ti,ab,kw OR (JAK Inhibitor):ti,ab,kw OR (Inhibitor, JAK):ti,ab,kw OR (Inhibitors, JAK):ti,ab,kw

#4 (JAK1):ti,ab,kw OR (JAK2):ti,ab,kw OR (JAK3):ti,ab,kw OR (TYK2):ti,ab,kw

#5 (abrocitinib):ti,ab,kw OR (PF-04965842):ti,ab,kw OR (PF04965842):ti,ab,kw

#6 (ritlecitinib):ti,ab,kw OR (PF-06651600):ti,ab,kw OR (PF06651600):ti,ab,kw

#7 (tofacitinib):ti,ab,kw OR (CP-690550):ti,ab,kw OR (CP690550):ti,ab,kw

#8 (baricitinib):ti,ab,kw OR (LY3009104):ti,ab,kw OR (LY-3009104):ti,ab,kw

#9 (ruxolitinib):ti,ab,kw OR (INCB018424):ti,ab,kw OR (INCB-018424):ti,ab,kw

#10 (upadacitinib):ti,ab,kw OR (ABT-494):ti,ab,kw OR (ABT494):ti,ab,kw

#11 (brepocitinib):ti,ab,kw OR (PF-06700841):ti,ab,kw OR (PF06700841):ti,ab,kw

#12 (fedratinib):ti,ab,kw OR (SAR302503):ti,ab,kw OR (SAR-302503):ti,ab,kw OR (TG101348):ti,ab,kw OR (TG-101348):ti,ab,kw

#13 (filgotinib):ti,ab,kw OR (GLPG-0634):ti,ab,kw OR (GLPG0634):ti,ab,kw

#14 (pacritinib):ti,ab,kw OR (SB1518):ti,ab,kw OR (SB-1518):ti,ab,kw

#15 (delgocitinib):ti,ab,kw OR (JTE-052):ti,ab,kw OR (JTE052):ti,ab,kw

#16 (peficitinib):ti,ab,kw OR (ASP015K):ti,ab,kw OR (JNJ-54781532):ti,ab,kw OR (JNJ54781532):ti,ab,kw

#17 (oclacitinib):ti,ab,kw OR (PF-03394197):ti,ab,kw OR (PF03394197):ti,ab,kw

#18 (nezulcitinib):ti,ab,kw OR (TD-0903):ti,ab,kw OR (TD0903):ti,ab,kw

#19 (deuruxolitinib):ti,ab,kw OR (CTP-543):ti,ab,kw OR (CTP543):ti,ab,kw

#20 (Ivarmacitinib):ti,ab,kw OR (SHR0302):ti,ab,kw OR (ARQ-255):ti,ab,kw OR (ARQ255):ti,ab,kw OR (ARQ-252):ti,ab,kw

#21 (jaktinib):ti,ab,kw OR (ARQ252):ti,ab,kw

#22 (ATI-501):ti,ab,kw OR (ATI501):ti,ab,kw OR (ATI-502):ti,ab,kw OR (ATI502):ti,ab,kw

#23 #1 OR #2 OR #3 OR #4 OR #5 OR #6 OR #7 OR #8 OR #9 OR #10 OR #11 OR #12 OR #13 OR #14 OR #15 OR #16 OR #17 OR #18 OR #19 OR #20 OR #21 OR #22

#24 MeSH descriptor: [Alopecia Areata] explode all trees

#25 (alopecia areata):ti,ab,kw OR (alopecia):ti,ab,kw OR (areata):ti,ab,kw OR (alopecia totalis):ti,ab,kw OR (alopecia universalis):ti,ab,kw

#26 (Hair Loss):ti,ab,kw OR (Hair Losses):ti,ab,kw OR (patchy hair loss):ti,ab,kw OR (Alopecia Circumscripta):ti,ab,kw

#27 #24 OR #25 OR #26

#28 #23 AND #27

***Web of Science***

#1 TS=(Janus Kinase Inhibitors) or TS=(Janus Kinase Inhibitor) or TS=(Inhibitors, Janus Kinase) or TS=(Kinase Inhibitors, Janus) or TS=(JAK Inhibitors) or TS=(Inhibitors, JAK) or TS=(Inhibitor, Janus Kinase) or TS=(Kinase Inhibitor, Janus) or TS=(JAK Inhibitor) or TS=(Inhibitor, JAK) or TS=(JAK1) or TS=(JAK2) or TS=(JAK3) or TS=(TYK2) or TS=(abrocitinib) or TS=(PF-04965842) or TS=(PF04965842) or TS=(ritlecitinib) or TS=(PF-06651600) or TS=(PF06651600) or TS=(tofacitinib) or TS=(CP-690550) or TS=(CP690550) or TS=(baricitinib) or TS=(LY3009104) or TS=(LY-3009104) or TS=(ruxolitinib) or TS=(INCB018424) or TS=(INCB-018424) or TS=(upadacitinib) or TS=(ABT-494) or TS=(ABT494) or TS=(brepocitinib) or TS=(PF-06700841) or TS=(PF06700841) or TS=(fedratinib) or TS=(SAR302503) or TS=(SAR-302503) or TS=(TG101348) or TS=(TG-101348) or TS=(filgotinib) or TS=(GLPG-0634) or TS=(GLPG0634) or TS=(pacritinib) or TS=(SB1518) or TS=(SB-1518) or TS=(delgocitinib) or TS=(JTE-052) or TS=(JTE052) or TS=(peficitinib) or TS=(ASP015K) or TS=(JNJ-54781532) or TS=(JNJ54781532) or TS=(oclacitinib) or TS=(PF-03394197) or TS=(PF03394197) or TS=(nezulcitinib) or TS=(TD-0903) or TS=(TD0903) or TS=(deuruxolitinib) or TS=(CTP543) or TS=(CTP-543) or TS=(ivarmacitinib) or TS=(ARQ-255) or TS=(ARQ255) or TS=(SHR0302) or TS=(ARQ-252) or TS=(ARQ252) or TS=(jaktinib) or TS=(ATI-501) or TS=(ATI501) or TS=(ATI-502) or TS=(ATI502)

#2 TS=(alopecia areata) or TS=(alopecia) or TS=(areata) or TS=(alopecia totalis) or TS=(alopecia universalis) or TS=(Hair Loss) or TS=(Hair Losses) or TS=(patchy hair loss) or TS=(alopecia circumscripta)

#3 #1 AND #2

**
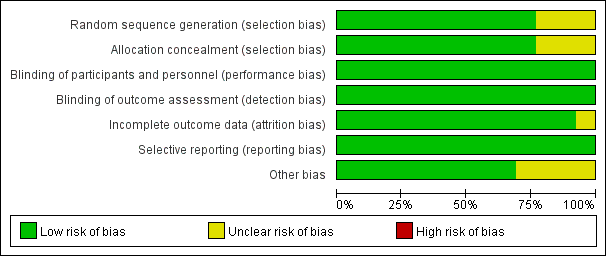
**

**Supplementary Figure 1** Risk of bias graph: review authors’ judgments about each risk of bias item presented as percentages across all included studies

**
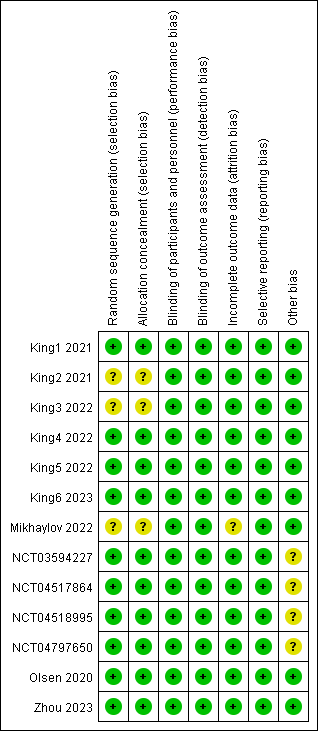
**

**Supplementary Figure 2** 'Risk of bias' summary: review authors' judgements about each 'Risk of bias' item for each included study

**(A)**


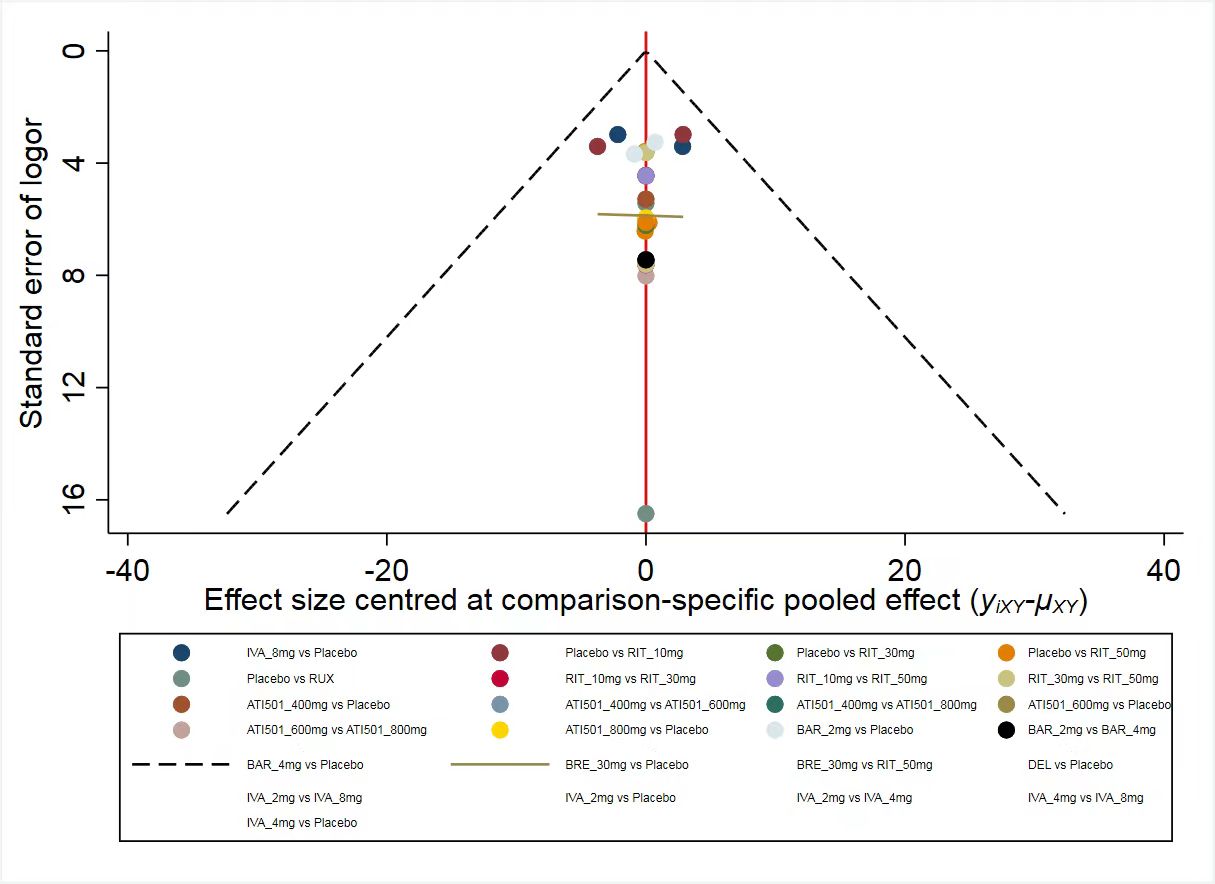


**(B)**

**
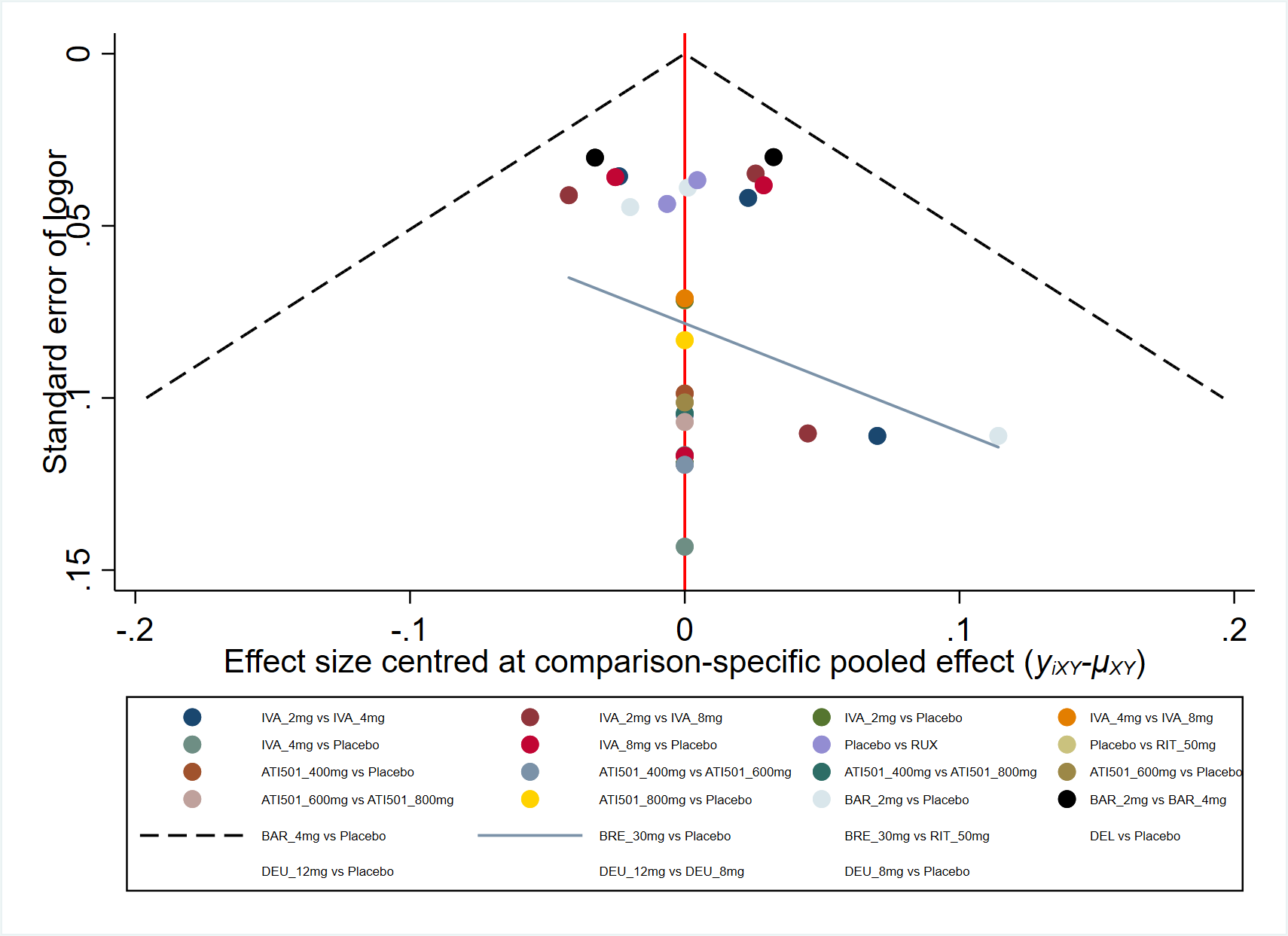
**

**(C)**


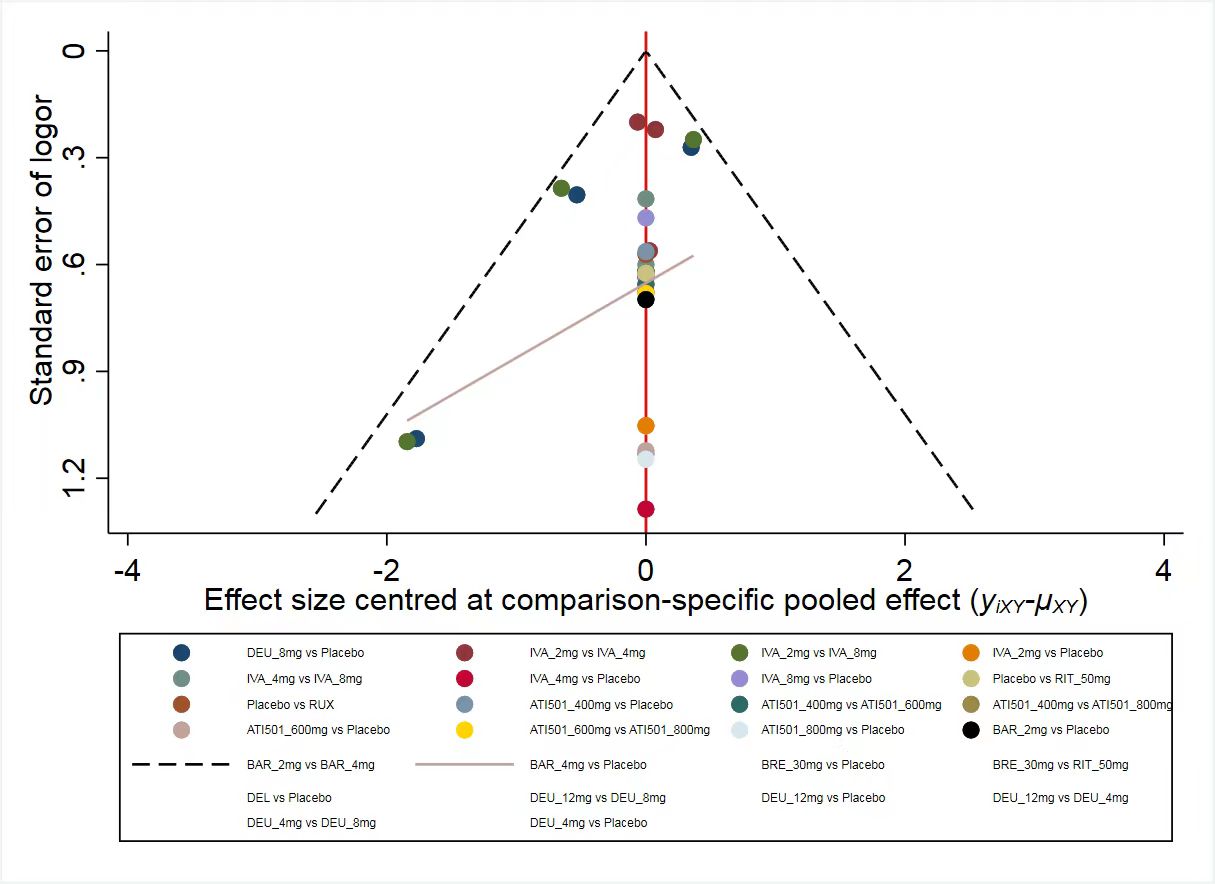


**(D)**

**
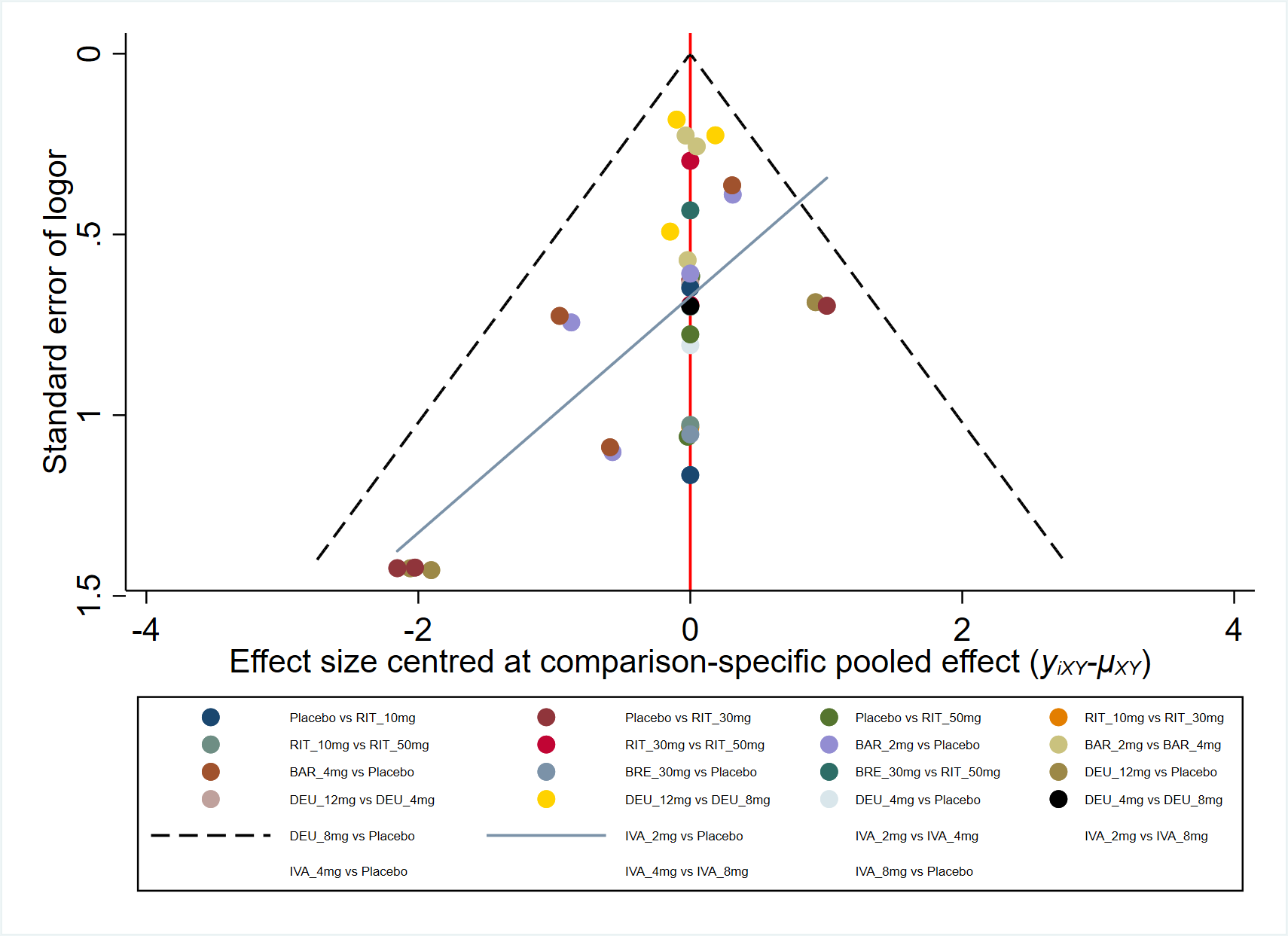
**

**(E)**

**
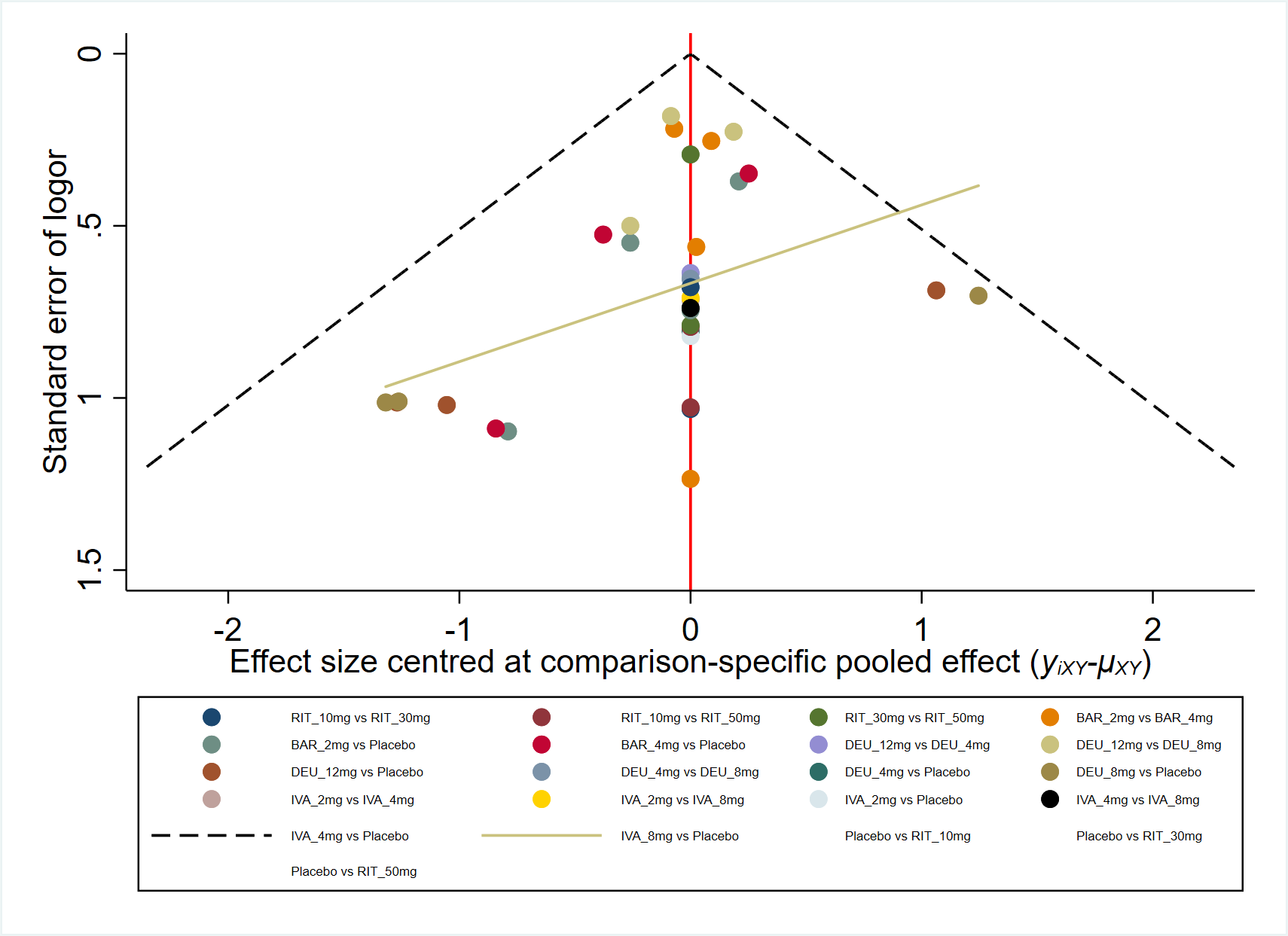
**

**(F)**

**
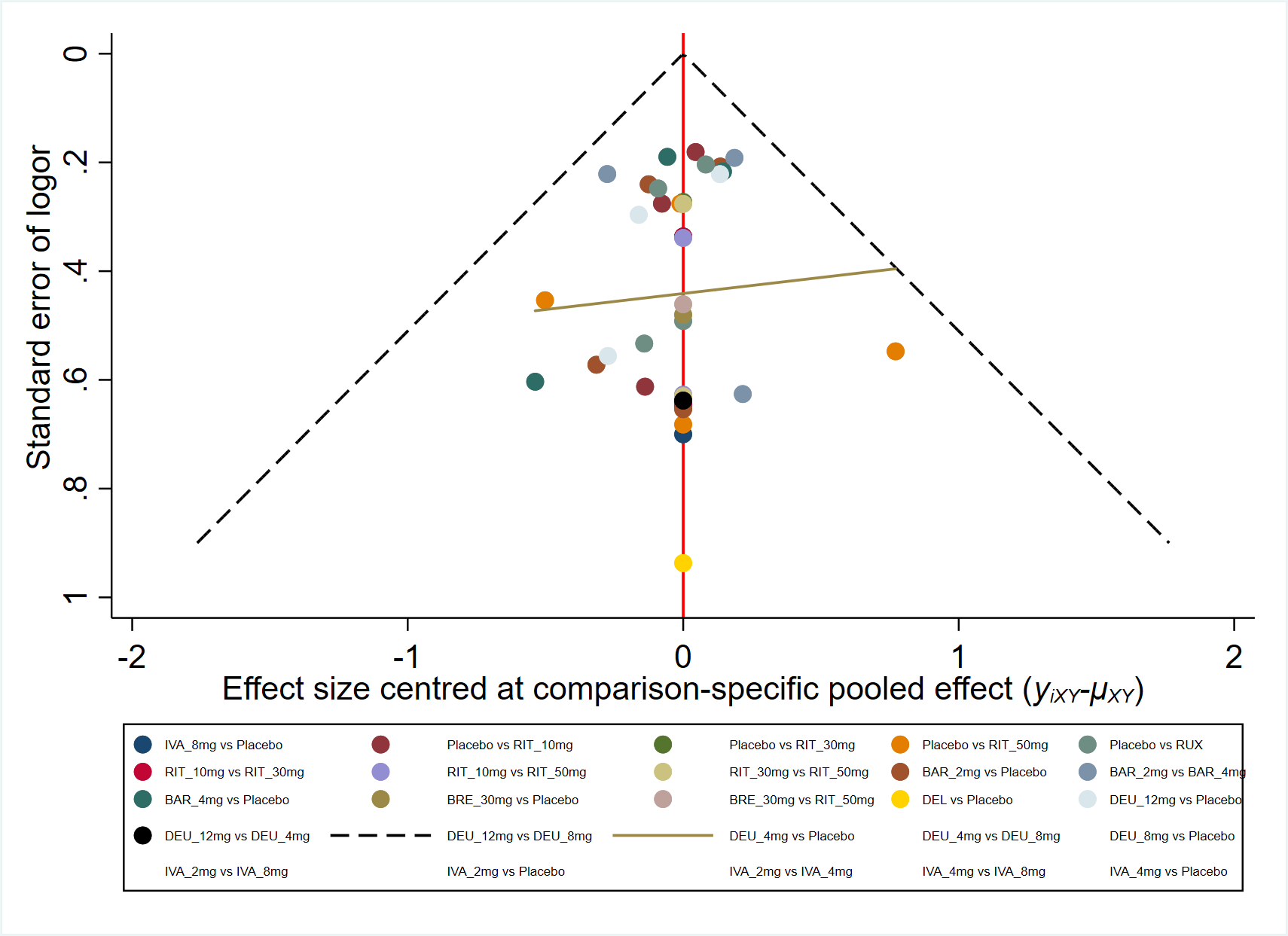
**

**Supplementary Figure 3** Comparison-adjusted funnel plot for outcome indicators of the network meta-analysis (A) the change in SALT score; (B) the percentage change in SALT score; (C) SALT_50_; (D) SALT_75_; (E) the percentage of patients who achieved SALT score≤20; (F) AEs (adverse events)
